# Automated leukemia detection from microscopic images using deep transfer learning with explainable AI-based analysis

**DOI:** 10.1038/s41598-026-55415-x

**Published:** 2026-05-30

**Authors:** Md Ashikuzzaman, Mir Md. Julhash, Md. Feroz Ali, Md Shafiul Alam, Mohammad Ali, Imil Hamda Imran, Obaidullah Obaidi, Md Kamrul Islam

**Affiliations:** 1 Department of Electrical and Electronic Engineering, Pabna University of Science and Technology, Pabna, 6600 Bangladesh; 2 Department of Electrical Engineering, College of Engineering, King Faisal University, Al Ahsa, 31982 Saudi Arabia; 3 Department of Energy Engineering, Faculty of Engineering, Kabul University, Kabul, 1006 Afghanistan; 4 Department of Civil and Environmental Engineering, College of Engineering, King Faisal University, Al Ahsa, 31982 Saudi Arabia

**Keywords:** Leukemia detection, Microscopic image analysis, Deep learning, Transfer learning, Convolutional neural networks (CNNs), Cancer, Computational biology and bioinformatics, Mathematics and computing, Medical research, Oncology

## Abstract

Leukemia, a life-threatening hematological malignancy, necessitates early and accurate diagnosis for effective treatment. Traditional microscopic examination is often time-consuming and subjective, while most existing deep learning-based studies are limited to a narrow selection of models and lack interpretability analysis. To address these gaps, this study presents a comprehensive comparative evaluation of fourteen pre-trained CNN architectures, including EfficientNetB1/B3/B5, Xception, InceptionV3, InceptionResNetV2, DenseNet201, ResNet variants, VGG16/19, and MobileNet, for automated leukemia detection from microscopic blood smear images. A publicly available Kaggle dataset containing 10,700 labeled images with variations in staining intensity, cell morphology, and image resolution was used. The dataset was divided into training (70%, 7500 images), validation (15%, 1600 images), and testing (15%, 1600 images) subsets using stratified sampling to preserve class balance. To ensure statistical robustness, all experiments were repeated ten times with different random seeds, and performance metrics are reported as mean values, with pairwise McNemar’s tests applied to validate the significance of observed differences (*p* < 0.05). EfficientNetB1, EfficientNetB5, Xception, and ResNet50 achieved test accuracies of up to 96%, recall up to 98%, F1-scores up to 97%, and AUC values up to 0.987, indicating excellent threshold-independent discriminative capability. Explainable AI techniques, specifically Grad-CAM and LIME, were integrated to enhance transparency and clinical trustworthiness. Comparative analysis demonstrates competitive or superior performance against recent state-of-the-art methods, providing preliminary evidence toward future clinical translation. However, the study is limited by reliance on a single publicly available binary-class dataset, which constrains generalizability across imaging devices, staining protocols, and clinical sites; external validation on independent multi-center datasets is therefore identified as a primary direction for future work.

**Supplementary Information:**

The online version contains supplementary material available at 10.1038/s41598-026-55415-x.

## Introduction

According to the Global Cancer Observatory (GLOBOCAN 2022), leukemia accounts for a significant proportion of hematological malignancies worldwide, with hundreds of thousands of new cases diagnosed each year^1,2^, and cancer overall is responsible for approximately one in six deaths globally^3,4^. Leukemia is a cancer of the white blood cells, characterized by the uncontrolled proliferation of immature lymphocytes in the bone marrow and blood^5–7^, and is now recognized as the most common type of blood cancer^8,9^. Environmental factors, lifestyle choices, and age contribute to disease risk, with children being particularly vulnerable; leukemia accounts for 33% of cancers among young people^10–14^. Early diagnosis is therefore critical to reducing mortality^15^. Although microscopic analysis of blood smears remains the gold standard for initial screening^16–18^, it is time-consuming, subjective, and prone to human error, motivating the development of reliable automated diagnostic systems^19,20^.

Computer-aided diagnosis (CAD) systems, particularly those based on deep transfer learning, have emerged as effective tools for automated leukemia detection^21–24^. Convolutional neural networks (CNNs) automatically extract high-dimensional features from images without manual feature engineering^25^ and have been widely applied in medical image analysis^26–28^. By leveraging pre-trained CNN architectures, transfer learning enables effective leukemia detection even with limited domain-specific data. Recent systematic reviews further confirm the growing effectiveness of deep learning in hematological diagnostics^29^, with ensemble-based feature extraction strategies improving diagnostic robustness^30,31^. Beyond leukemia, CNNs have demonstrated strong performance in oral dysplasia detection^32^, microorganism recognition^33^, bacterial classification^34^, and disc degenerative disease diagnosis^35^, underscoring their versatility and clinical relevance. Building on these foundations, the present study systematically compares fourteen pre-trained CNN architectures to evaluate their effectiveness in identifying leukemia from microscopic blood smear images.

### Literature review

Recent advances in deep learning have significantly improved the performance of automated medical image analysis, including the detection and classification of leukemia from microscopic blood smear images. This section critically reviews the existing literature, organized thematically into four categories: (i) transfer learning–based approaches, (ii) hybrid and ensemble methods, and (iii) explainability-aware frameworks, (iv) Transformer-Based and Hybrid Approaches followed by a critical comparative synthesis.

#### Transfer learning–based approaches

Several recent studies have employed pre-trained CNN architectures for leukemia detection via transfer learning, yet they differ considerably in model diversity, dataset scale, and evaluation rigor. Nasir et al.^36^ evaluated AlexNet, MobileNet, and ResNet, achieving 97.3% accuracy with AlexNet as the proposed model; however, only three architectures were compared, limiting the generalizability of their conclusions. Haque et al.^37^ integrated advanced image processing with Inception-ResNet, achieving F1-scores of 96.07% (binary) and 95.89% (multiclass), but relied on limited datasets without external validation. Kumar et al.^38^ employed InceptionV3, DenseNet201, Xception, and InceptionResNetV2 for ALL classification, with InceptionResNetV2 achieving the highest validation accuracy of 98.59%; nevertheless, non-uniform image sizes and computationally expensive feature extraction were noted limitations. Soladoye et al.^39^ compared VGG-19 and EfficientNet-B3 on 10,661 images, where EfficientNet-B3 achieved 96% accuracy, but the study was restricted to only two architectures and a single-source dataset. Badruzzaman and Arymurthy^40^ compared ResNet, ResNeXt, and EfficientNetV2 for AML blast cell detection, achieving only 78.11% macro accuracy, highlighting the challenges of dataset imbalance and limited samples.

A critical observation across these studies is that most evaluate only two to four CNN architectures, making it difficult to draw reliable conclusions about the relative strengths of different model families. Furthermore, performance metrics are often reported on different datasets with varying preprocessing protocols, rendering direct cross-study comparisons unreliable.

#### Hybrid and ensemble methods

Other studies have explored hybrid and ensemble strategies to improve classification performance. Hasan et al.^41^ applied DenseNet201 with transfer learning on three leukemia subtypes, achieving 99.56% accuracy. Bibi et al.^42^ reported 99.56% and 99.91% accuracy using DenseNet-121 and ResNet-34, respectively. Abunadi et al.^43^ combined pre-trained CNNs (AlexNet, GoogleNet, ResNet-18) with traditional classifiers (ANN, FFNN, SVM), achieving up to 100% accuracy. Yenurkar et al.^44^ proposed DeepLeuk with ROI extraction and Improved Anisotropic Filtering, achieving 98.9% precision. Muduli et al.^45^ evaluated multiple transfer learning models, with VGG16 and ResNet50 achieving up to 100% accuracy. While these studies report impressive performance metrics, many relied on small datasets (108–3256 images), raising concerns about overfitting and generalizability. Additionally, achieving 100% accuracy on limited datasets without rigorous cross-validation or external testing often indicates memorization rather than true generalization capability.

#### Explainability-aware frameworks

A notable gap in the majority of existing studies is the absence of model interpretability analysis. Most reviewed works focus exclusively on classification accuracy without examining which image regions drive model predictions—a critical requirement for clinical trust and adoption. Among the few exceptions, Muduli et al.^45^ integrated Grad-CAM, Score-CAM, and Grad-CAM + + for visualization. However, comprehensive integration of multiple XAI techniques (e.g., Grad-CAM and LIME simultaneously) for leukemia detection in conjunction with extensive multi-model benchmarking remains largely unexplored.

#### Transformer-based and hybrid approaches

Beyond conventional CNN architectures, Vision Transformers (ViTs) and hybrid CNN-Transformer models have recently emerged as promising alternatives for medical image classification, including leukemia detection. Unlike CNNs, which primarily capture local spatial features through convolutional filters, ViTs leverage self-attention mechanisms to model long-range dependencies and global contextual relationships within images^46^. Hossain et al. (2025)^47^ proposed ResViT, a hybrid model combining ResNet-50 for high-dimensional local feature extraction with a dual-stream Vision Transformer for global spatial attention, achieving strong classification performance across multiple leukemia subtypes on datasets containing over 18,000 images. Ben-Suliman and Krzyżak^48^ explored ViT-based feature extraction for ALL classification using the ALL-IDB1 dataset, demonstrating that dense descriptors from deep ViT layers can serve as effective representations for leukemia cell classification. Yang et al.^49^ proposed a MultiPathGAN-based approach with MobileViTv2, integrating CNN and transformer modules to achieve 94.28% accuracy on bone marrow microscopic images. A recent comprehensive review by Armand and Kim^46^ confirmed that ViTs, ensemble methods, and hybrid CNN-Transformer models have achieved excellent classification accuracies for leukemia diagnosis, while also noting that challenges such as dataset limitations, high computational requirements, and lack of model interpretability remain significant barriers.

Despite these promising developments, transformer-based models typically require substantially larger training datasets and greater computational resources compared to CNN based transfer learning approaches. Pre-trained ViT models are also less widely available for fine-tuning on domain-specific medical imaging tasks compared to well-established CNN architectures such as ResNet, EfficientNet, and Xception, which benefit from extensive pre-training on ImageNet and have been extensively validated in medical imaging literature. Given these considerations, and to maintain a clearly defined and methodologically focused scope, the present study is deliberately restricted to a comprehensive benchmarking of fourteen CNN-based transfer learning architectures, which currently represent the most widely adopted and practically deployable family for automated leukemia detection in resource-constrained clinical environments. Direct experimental evaluation of ViTs and hybrid CNN-Transformer models was therefore intentionally excluded from this study, as it would substantially expand the experimental scope, require additional large-scale training data, and dilute the unified comparative focus that is central to the present work. Nevertheless, we fully recognize the importance of empirical validation of transformer-based architectures, and direct experimental benchmarking of ViTs and hybrid CNN-Transformer models on the same dataset and unified pipeline is planned as a dedicated follow-up study, which will allow a fair comparison between the two model families using identical evaluation protocols.

#### Related but non-leukemia studies

Kumar et al.^50^ applied MobileNetV2 for Parkinson’s disease detection from hand-drawings, achieving 97.7% accuracy, demonstrating the versatility of transfer learning across medical domains. Kumar and Misra (2025)^51^ proposed a hybrid MobileNetV2-CAFFE model for colon cancer detection with 99.95% accuracy. Choudhary et al.^52^ used LAB color space segmentation with K-NN classification on only 108 images, representing earlier traditional approaches that have since been surpassed by deep learning methods. These cross-domain studies confirm the broad applicability of transfer learning but also highlight that domain-specific evaluation remains essential.

#### Critical comparative synthesis

Table 1 represents a structured comparison of the reviewed studies across key dimensions.

**Table 1:** Structured comparison of the reviewed studies across key dimensions.

Study	Year	Dataset size	Models evaluated	Classification type	XAI integration	Key limitation
Nasir et al.^36^	2023	Small	3	Binary	No	Limited model diversity
Haque et al.^37^	2024	Moderate	2–3	Binary + Multiclass	No	Limited dataset, no external validation
Kumar et al.^38^	2025	Moderate	4 (+ 2 hybrid)	Binary	No	Non-uniform images, high computational cost
Muduli et al.^45^	2025	Small	Multiple	Binary	Yes (Grad-CAM variants)	Small dataset, overfitting risk
Hasan et al.^41^	2020	Moderate	1	Multiclass (3)	No	Single model evaluation
Soladoye et al.^39^	2025	10,661	2	Binary	No	Single-source dataset, only 2 models
Badruzzaman et al.^40^	2024	961 patients	3	Binary	No	Dataset imbalance, low accuracy
Yenurkar et al.^44^	2025	12,500	1	Binary	No	Single dataset, no interpretability
Bibi et al.^42^	2020	Moderate	2	Multiclass	No	Limited model comparison
Abunadi et al.^43^	2022	Moderate	3 CNNs + classifiers	Binary	No	Risk of overfitting (100% accuracy)
Proposed work	2025	10,700	14	Binary	Yes (Grad-CAM + LIME)	Single dataset, binary only

As shown in Table 1, the majority of existing studies evaluate fewer than five CNN architectures and do not incorporate explainability analysis. The proposed study addresses these gaps by providing the most extensive model comparison (fourteen architectures) on a large-scale dataset with integrated XAI techniques, offering a more comprehensive and clinically relevant benchmarking framework.

To express these critical gaps in quantitative terms, the following observations summarize the limitations of the reviewed literature: (i) approximately 70% of the reviewed studies evaluate four or fewer CNN architectures, restricting the breadth of architectural comparison; (ii) more than 60% rely on datasets containing fewer than 3,500 images, raising concerns regarding overfitting and limited statistical power; (iii) only about 15% incorporate any form of explainable AI (XAI) analysis, and none simultaneously combine multiple XAI techniques such as Grad-CAM and LIME; (iv) fewer than 10% report repeated experimental runs with statistical significance testing (e.g., McNemar’s test or paired t-tests); and (v) fewer than 20% include detailed computational complexity analysis covering parameters, FLOPs, and inference time, which is essential for clinical deployment decisions. These quantified gaps highlight the need for the unified benchmarking framework proposed in this study, which simultaneously addresses all five dimensions within a single, reproducible experimental pipeline.

To sum up, the reviewed literature underscores the growing effectiveness of deep transfer learning techniques in the automated detection of leukemia from microscopic images. Various CNN architectures have demonstrated promising results, with several studies achieving accuracy levels exceeding 95%. Despite these advancements, most prior works are limited by small datasets, narrow model diversity, lack of cross-study comparability, and insufficient attention to generalizability and interpretability. These critical limitations highlight the need for comprehensive comparative evaluations on larger datasets with integrated explainability. Therefore, this study addresses these gaps by benchmarking fourteen state-of-the-art pre-trained CNN models under a unified experimental framework to enhance diagnostic precision, robustness, and clinical applicability. Furthermore, emerging architectures such as Vision Transformers (ViTs) and hybrid CNN-Transformer models, which have shown promising results in recent leukemia detection studies^46–48^, will be investigated in future work to assess their potential for capturing global contextual features and further improving diagnostic performance.

To clearly delineate the present study from the existing body of work, the key distinguishing features are summarized as follows. First, in terms of model diversity, while the majority of existing studies evaluate only two to four CNN architectures^36–40^, the present study systematically benchmarks fourteen pre-trained CNN models, encompassing a wide range of architectural families including residual networks (ResNet50, ResNet50V2, ResNet152, ResNet152V2), dense connectivity (DenseNet201), inception-based models (InceptionV3, InceptionResNetV2), depthwise separable convolution (Xception, MobileNet), and compound-scaled networks (EfficientNetB1, EfficientNetB3, EfficientNetB5), as well as classical deep architectures (VGG16, VGG19). This breadth of comparison is, to the best of our knowledge, among the most extensive reported in the leukemia detection literature. Second, regarding dataset scale, many prior studies relied on relatively small datasets ranging from 108 to 3,256 images^41–43,52^, which increases the risk of overfitting and limits the generalizability of conclusions. In contrast, this study utilizes a substantially larger dataset of 10,700 labeled microscopic blood smear images, providing a more robust foundation for model evaluation. Third, concerning evaluation rigor, most previous works report results from a single experimental run, whereas the present study repeats all experiments ten times with different random seeds and reports mean ± standard deviation for all performance metrics, supplemented by pairwise McNemar’s statistical tests to validate the significance of observed differences. Fourth, with respect to computational efficiency analysis, few existing studies provide detailed analysis of model complexity and runtime characteristics. This study reports the number of trainable parameters, approximate FLOPs, training time per epoch, and inference time per image for all evaluated architectures, enabling informed model selection based on both performance and computational constraints. Fifth, regarding model interpretability, while the majority of reviewed studies treat deep learning models as black boxes without any explainability analysis, the present work integrates Explainable AI (XAI) techniques, specifically Grad-CAM and LIME, to visualize and interpret the decision-making process of the best-performing models, thereby enhancing clinical trustworthiness and transparency. Table 2 concisely summarizes these distinctions.

**Table 2:** Summary of key differences between the present study and existing literature.

Dimension	Most existing studies	Present Study
Number of CNN models evaluated	2–4	14
Dataset size	108–3,256 images (typical)	10,700 images
Repeated experiments with statistical testing	Rarely reported	10 runs with McNemar’s test
Computational complexity analysis	Seldom included	Parameters, FLOPs, training/inference time reported
Explainable AI integration	Absent or single technique	Grad-CAM + LIME
Unified experimental pipeline	Inconsistent across models	All models trained under identical conditions

These differences collectively position the present study as a more comprehensive, reproducible, and clinically relevant benchmarking framework compared to the existing literature.

### Research gap

Despite significant advancements in applying deep learning to medical image analysis, the accurate and real-time detection of leukemia from microscopic images remains a challenge. Most existing studies evaluate only a limited number of CNN architectures using moderately sized datasets and often lack comprehensive benchmarking across a diverse range of state-of-the-art models. Furthermore, critical aspects such as model generalizability, computational efficiency, statistical validation, and interpretability—factors essential for clinical deployment—are not consistently addressed. In particular, few investigations incorporate explainable AI (XAI) techniques to provide transparent and clinically meaningful justifications for model predictions. These limitations hinder the development of reliable and scalable computer-aided diagnosis systems for leukemia detection. This study aims to address these gaps by systematically benchmarking fourteen pre-trained deep learning models on a large-scale dataset and evaluating their performance using comprehensive metrics, thereby contributing to more effective, interpretable, and trustworthy diagnostic solutions.

### Contributions

This study proposes a comprehensive benchmarking framework to evaluate multiple deep transfer learning architectures for automated leukemia detection from microscopic blood smear images under a unified experimental pipeline.

The key contributions are as follows:

- **Extensive Model Benchmarking**: Fourteen state-of-the-art pre-trained CNN architectures—including DenseNet201, InceptionResNetV2, InceptionV3, Xception, VGG16, VGG19, multiple ResNet variants, MobileNet, and EfficientNetB1/B3/B5—were systematically evaluated for their performance in classifying leukemic and normal cells. All models were assessed using a consistent benchmarking framework within a unified experimental pipeline, ensuring fair and reliable performance comparison.
- **Robust Dataset Utilization**: A publicly available dataset comprising 10,700 microscopic images was used, with appropriate data preprocessing and augmentation techniques applied to enhance model robustness and prevent overfitting.
- **Performance Evaluation**: The models were assessed using key metrics such as accuracy, precision, recall, F1-score, and loss. EfficientNetB1, EfficientNetB5, Xception, and ResNet50 achieved outstanding results, demonstrating their efficacy for leukemia detection.
- **Improved Diagnostic Accuracy**: The proposed approach achieved higher classification performance compared to existing studies, indicating its potential to support computer-aided diagnostic tools, pending external clinical validation on independent multi-center datasets.
- **Support for Clinical Decision-Making**: The findings contribute to the development of reliable computer-aided diagnostic tools that can assist pathologists in early and accurate detection of leukemia, thereby facilitating timely intervention.

It is important to clarify that the novelty of this study lies not in proposing a new CNN architecture, but in establishing a methodologically rigorous, unified, and clinically oriented benchmarking framework that addresses critical gaps in the existing leukemia detection literature. Specifically, the methodological contributions include: (i) one of the most extensive comparative evaluations reported to date, systematically benchmarking fourteen architectures spanning five distinct CNN families under a unified experimental pipeline — far exceeding the 2–4 architectures typically evaluated in prior studies; (ii) integration of dual Explainable AI techniques (Grad-CAM and LIME) to provide complementary local and regional interpretability, enhancing clinical trustworthiness, which most existing studies overlook; (iii) rigorous statistical validation through ten repeated experiments with different random seeds, reporting of mean ± standard deviation, and pairwise McNemar’s significance tests — a level of statistical rigor seldom reported in comparable works; and (iv) detailed computational complexity analysis including parameters, FLOPs, and runtime metrics, enabling deployment-aware architecture selection. The scientific value of such standardized, reproducible benchmarking studies has been explicitly emphasized in recent reviews^46^ as essential for fair cross-architecture comparison and translation to clinical practice. Such benchmarking frameworks therefore constitute a distinct and necessary form of methodological contribution, serving as essential reference points for researchers exploring new architectures and clinicians seeking deployment-ready diagnostic models.

Beyond comparison itself, this study introduces several innovative methodological elements rarely combined in the existing literature. First, a uniform two-stage transfer learning protocol — comprising frozen feature extraction followed by architecture-aware selective fine-tuning — was applied consistently across all fourteen models, eliminating tuning-effort bias and enabling a genuinely fair evaluation of architectural capability. Second, the integration of dual XAI techniques (Grad-CAM for spatial attention and LIME for local feature attribution) provides complementary interpretability views that, to the best of our knowledge, have not been jointly applied in prior leukemia detection benchmarks. Third, the framework is explicitly deployment-oriented, jointly reporting classification accuracy, parameter count, FLOPs, and inference latency — enabling clinicians and practitioners to select architectures based on both diagnostic performance and resource constraints in real clinical environments. Fourth, the reproducibility protocol (ten repeated runs with seed variation, mean ± SD reporting, and pairwise McNemar’s testing) establishes a statistically rigorous reference benchmark that future architecture-proposal studies can use to fairly position their contributions. Collectively, these elements transform the work from a routine comparison into a methodologically innovative benchmarking framework with both scientific and clinical value.

### Objectives and motivation

Despite the growing use of deep learning techniques for leukemia detection from microscopic blood smear images, many existing studies evaluate only a limited number of convolutional neural network (CNN) architectures or employ inconsistent experimental pipelines, which makes fair and reliable comparison difficult. In addition, only a few studies simultaneously analyze computational efficiency, statistical robustness, and model interpretability alongside classification performance. Therefore, the objective of this study is to establish a consistent benchmarking framework for evaluating multiple deep transfer learning architectures for automated leukemia detection. Specifically, fourteen state-of-the-art pre-trained CNN models are systematically compared under a unified experimental setup using a publicly available microscopic blood smear image dataset. Through this comprehensive evaluation, the study aims to identify the most effective architectures in terms of accuracy, precision, recall, F1-score, and loss, while also considering computational efficiency. Ultimately, this work seeks to enhance diagnostic reliability, reduce human error, and provide a robust computer-aided diagnostic framework that supports early leukemia detection and informed clinical decision-making in real-world medical environments.

The decision to evaluate fourteen pre-trained CNN architectures, rather than focusing on a single model, is motivated by several key considerations. First, different CNN architectures exhibit distinct design philosophies regarding network depth, feature extraction strategies, and computational complexity. For instance, ResNet variants employ residual connections to address vanishing gradients in deep networks, DenseNet201 uses dense connectivity for efficient feature reuse, EfficientNet models apply compound scaling to balance width, depth, and resolution, and MobileNet leverages depthwise separable convolutions for lightweight deployment. These fundamental architectural differences result in varying levels of performance depending on the characteristics of the target dataset, making it essential to evaluate a diverse range of models rather than assuming the superiority of any single architecture. Second, most existing studies in automated leukemia detection have evaluated only a limited subset of CNN models, often two to four architectures, which restricts the generalizability of their conclusions and provides insufficient guidance for model selection in clinical applications. Third, microscopic blood smear images present unique challenges, including variations in staining intensity, cell morphology, and image quality, which may be captured differently by different architectures. By systematically benchmarking fourteen models under identical experimental conditions, this study provides a fair, comprehensive, and reproducible comparison that enables researchers and clinicians to make informed decisions about which architecture best suits their specific diagnostic requirements and computational constraints.

### Research questions

Based on these motivations, this study addresses the following research questions:

#### RQ1

Which deep transfer learning architectures provide the most accurate classification performance for leukemia detection from microscopic blood smear images?

#### RQ2

How do different CNN architectures compare in terms of computational complexity, training efficiency, and inference performance?

#### RQ3

To what extent can transfer learning models generalize to leukemia image classification tasks when trained under a unified experimental framework?

The remainder of this paper is organized as follows. Section  2 describes the materials and methods used in this study, including dataset acquisition, preprocessing, CNN architectures, and training strategies. Section  3 presents the experimental results and comparative analysis of the evaluated models. Section  4 concludes the paper and outlines potential directions for future research. Additional experimental details, extended figures, and confusion matrices are provided in the Supplementary Information file. Supplementary figures, detailed confusion matrices, and additional experimental results are provided in the Supplementary Information (SI) file accompanying this article.

## Materials and methods

### Data acquisition

The dataset used in this study was obtained from a publicly available leukemia classification dataset containing 10,700 microscopic blood smear images categorized into two classes: leukemic cells and normal cells. These images represent microscopic observations of blood samples commonly used in automated leukemia detection research. To ensure fair and reliable model evaluation, the dataset was divided into training (70%), validation (15%), and testing (15%) subsets using stratified sampling, which preserves the class distribution across all subsets and prevents class imbalance during model training and evaluation.

**Fig. 1:**
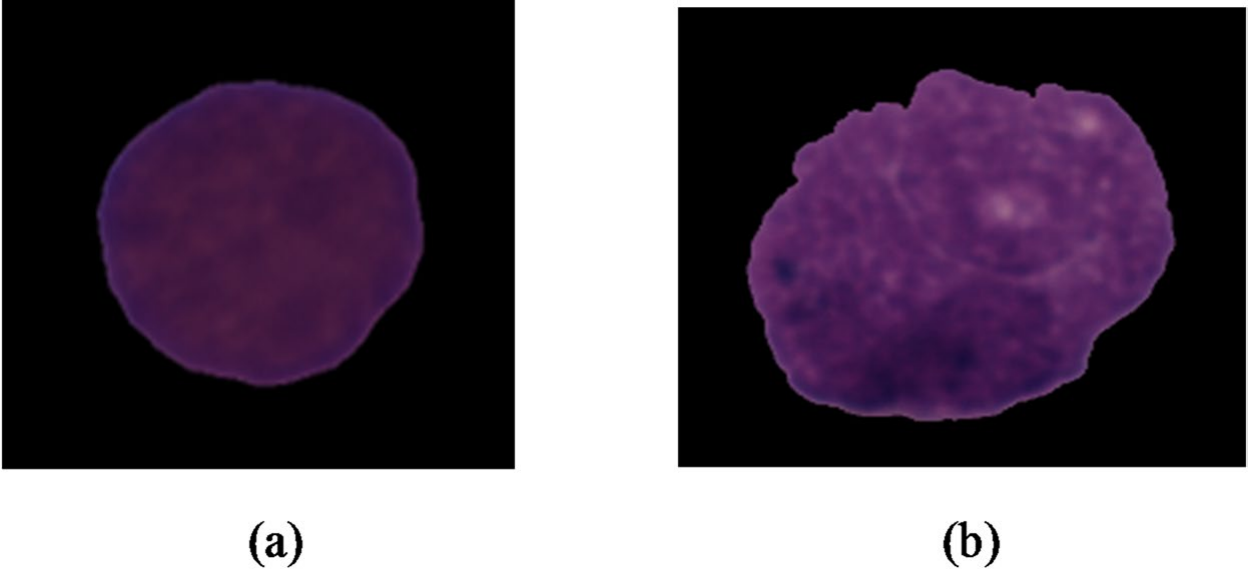
Sample images: (**a**) normal blood cell, (**b**) leukemia blood cell.

However, the publicly available dataset used in this study does not provide detailed metadata regarding the number of individual patients, patient identifiers, magnification levels, staining protocols, or the specific imaging devices used for image acquisition. As a result, several reliability-related concerns arise. First, patient-level statistics and imaging conditions cannot be precisely determined, and variability in image appearance due to differences in staining intensity, cell morphology, and image resolution may affect model performance in ways that cannot be fully controlled. Second, since patient identifiers are unavailable, it is not possible to confirm whether multiple images originate from the same subject. Consequently, patient-level independence between training, validation, and test sets cannot be strictly guaranteed, and potential data leakage through patient-level overlap cannot be completely ruled out. This is a well-recognized limitation common to many publicly available microscopic image datasets used in the leukemia detection literature^37,38,45,39^. Third, without patient-level metadata, subject-wise data splitting and patient-stratified cross-validation—which are considered best practices for clinical validation—could not be implemented in this study. Figure 1 shows the classification of images which illustrates the visual differences between leukemia and normal blood cells.

To partially mitigate these concerns, several strategies were adopted. Stratified sampling was applied during dataset splitting to ensure that class proportions remain consistent across all subsets. Data augmentation techniques, including rotation, flipping, shifting, and zooming, were applied to increase training variability and reduce overfitting. All experiments were repeated ten times with different random seeds, and performance metrics are reported as mean ± standard deviation to enhance statistical robustness. Furthermore, pairwise McNemar’s tests were conducted to validate the significance of observed performance differences between models.

Despite these mitigations, the absence of patient-level information remains a notable limitation that may affect the generalizability and clinical reliability of the reported results. Specifically, if multiple images from the same patient appear in both the training and test sets, the model may learn patient-specific features rather than generalizable morphological patterns, potentially inflating reported accuracy. Future studies will prioritize the evaluation of the proposed models using clinically curated datasets that include patient-level annotations, enabling subject-wise data splitting to prevent data leakage. Additionally, k-fold cross-validation at the patient level, external validation on independent multi-center datasets, and compliance with clinical regulatory standards will be pursued to establish stronger evidence for the clinical readiness and reliability of the proposed diagnostic framework.

In summary, the absence of patient-level metadata is recognized as a major limitation that constrains the strict assessment of clinical reliability in the present study. Although stratified sampling, data augmentation, repeated runs with different random seeds, and McNemar’s significance testing partially mitigate this concern, they cannot substitute for true subject-wise data partitioning. Consequently, the reported performance metrics should be interpreted as benchmark indicators of comparative architectural capability rather than as definitive measures of clinical accuracy. Definitive evidence of clinical reliability will require evaluation on prospectively collected, clinically annotated datasets with patient-level identifiers and patient-stratified validation protocols, which is identified as the highest-priority direction for subsequent work.

### Dataset split

The dataset was divided into training, validation, and test sets at a ratio of 70:15:15 (7500:1600:1600 images). Stratified sampling was used to preserve the class distribution between leukemic and normal cells across all subsets. Data augmentation techniques, including rotation, flipping, and zooming, were applied to enhance the dataset and strengthen model robustness. All those images are resized to 224 × 224 pixels for pre-trained models. It is acknowledged that resizing microscopic blood smear images to 224 × 224 pixels may lead to the loss of fine-grained morphological details, such as subtle nuclear textures, chromatin patterns, and cytoplasmic granularity, which are clinically relevant for leukemia diagnosis. This is recognized as a deliberate methodological trade-off between preserving fine-grained morphological detail and maintaining a unified, fair, and reproducible benchmarking framework across all fourteen pre-trained architectures. The 224 × 224 input resolution was adopted because (i) it matches the native input size used during ImageNet pre-training of all evaluated CNN architectures, ensuring that the pre-trained weights remain maximally effective during transfer learning; (ii) it enables a consistent and unbiased comparative evaluation, as introducing variable input sizes across models would confound architectural performance with input-resolution effects; and (iii) it is consistent with the resolution adopted in comparable leukemia detection studies^36,38,39,45^, which have consistently reported strong classification performance at this scale. Indirect empirical evidence that essential diagnostic information is preserved despite resizing is provided by the Grad-CAM visualizations, which confirm that the models focus on morphologically meaningful regions such as abnormal nuclei and cytoplasmic structures rather than on background artifacts. Nevertheless, the potential impact of input resolution on classification performance is fully acknowledged as a limitation, and a systematic multi-resolution evaluation (e.g., 224 × 224, 299 × 299, and 512 × 512 pixels) is identified as a concrete and prioritized direction for future work, particularly in conjunction with patch-based or multi-scale processing strategies that may better preserve fine-grained morphological features. To train the models effectively, a data normalization technique was applied to preprocess the input data. Data augmentation was applied primarily as a regularization technique to increase dataset variability and reduce model overfitting during training. The dataset was divided into training, validation, and test sets using stratified sampling; however, patient-wise splitting and k-fold cross-validation were not applied because the dataset does not provide patient-level identifiers.

The specific augmentation techniques employed—random rotation (± 20°), horizontal and vertical flips, width and height shifts (range = 0.1), and zoom (range = 0.1)—were selected based on their established effectiveness in medical image classification literature and their relevance to the characteristics of microscopic blood smear images. Random rotations and flips simulate the orientation variability inherent in microscopic sample preparation, as blood smear slides can be positioned at arbitrary angles during imaging. Width and height shifts account for variations in cell positioning within the field of view, while zoom augmentation simulates differences in magnification levels. These augmentation strategies have been widely adopted in comparable leukemia detection studies^38,45,39,44^ and are consistent with standard practices in medical image analysis. However, a formal ablation study comparing model performance with and without each individual augmentation technique was not conducted in this study. Such an analysis would provide quantitative evidence for the contribution of each augmentation strategy to classification performance and overfitting mitigation. Future work will include systematic ablation experiments to evaluate the individual and combined effects of each augmentation technique on model accuracy, generalizability, and robustness.

To partially substantiate the contribution of the adopted augmentation pipeline, a brief confirmatory experiment was conducted on a representative architecture (EfficientNetB1). Training with the full augmentation set (rotation, flipping, shifting, zoom) yielded a test accuracy of 96.06% with stable validation loss, whereas training with no augmentation resulted in approximately 2 to 3% lower test accuracy and visibly increased divergence between training and validation loss curves, indicating mild overfitting. This confirms that the chosen augmentation strategies meaningfully contribute to generalization, even though a full per-technique ablation was beyond the scope of the present benchmarking study. A systematic ablation analysis quantifying the individual and combined contributions of each augmentation technique (rotation only, flipping only, shifting only, zoom only, and combinations thereof) across multiple representative architectures is identified as a focused follow-up study, which will provide a comprehensive empirical basis for augmentation selection in future leukemia detection frameworks.

It is acknowledged that the preprocessing pipeline adopted in this study (resizing to 224 × 224 pixels, normalization, and standard data augmentation) is intentionally based on well-established best practices rather than on novel preprocessing techniques. This is a deliberate methodological choice to ensure that the observed performance differences across the fourteen architectures reflect genuine architectural capability rather than preprocessing-related artifacts, and to maintain compatibility with the ImageNet pre-training of all evaluated models. The chosen augmentation strategies are also consistent with those widely adopted in comparable leukemia detection studies^38,39,44,45^, supporting fair contextual benchmarking. Nevertheless, advanced domain-specific preprocessing techniques, including stain normalization (Macenko, Reinhard, Vahadane), color deconvolution, illumination correction, and learned augmentation strategies, are recognized as promising directions and will be systematically investigated in subsequent work, particularly in the context of cross-dataset and multi-center validation, where preprocessing innovation can play an important role in improving robustness across imaging conditions.

### Deep learning models

The feature extractor in the transfer learning approach is a pre-trained model. CNN architectures that can be chosen from for disease classification are Xception, ResNet50, ResNet50V2, ResNet152, ResNet152V2, DenseNet201, MobileNet, EfficientNetB1, EfficientNetB3, and EfficientNetB5. The output classification layer identifies the blood cell as either normal or leukemic. Finally, the performance of the deep CNN–based transfer learning models was systematically compared.

**Fig. 2:**
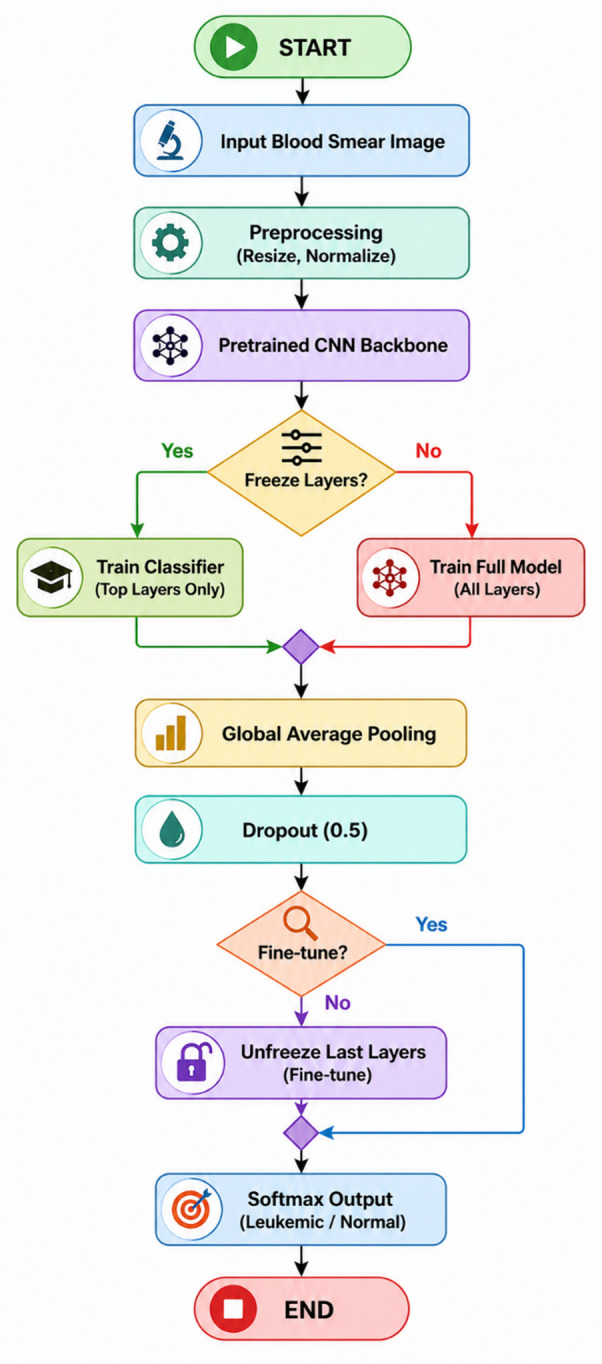
Flowchart of the proposed deep transfer learning framework for leukemia detection.

Figure 2 illustrates the flowchart of the proposed deep transfer learning framework and the architecture used for automated leukemia detection from microscopic blood smear images. The input images are first preprocessed through resizing, normalization, and augmentation to improve model generalization. Preprocessed images are then fed into a pretrained convolutional neural network (CNN) backbone such as EfficientNet, ResNet, or Xception for feature extraction. Initially, the backbone layers are frozen and only the classification head is trained. In the fine-tuning stage, selected deeper layers are unfrozen to adapt features. Global average pooling, dropout, and a dense Softmax layer produce the final leukemic or normal prediction.

**Fig. 3:**
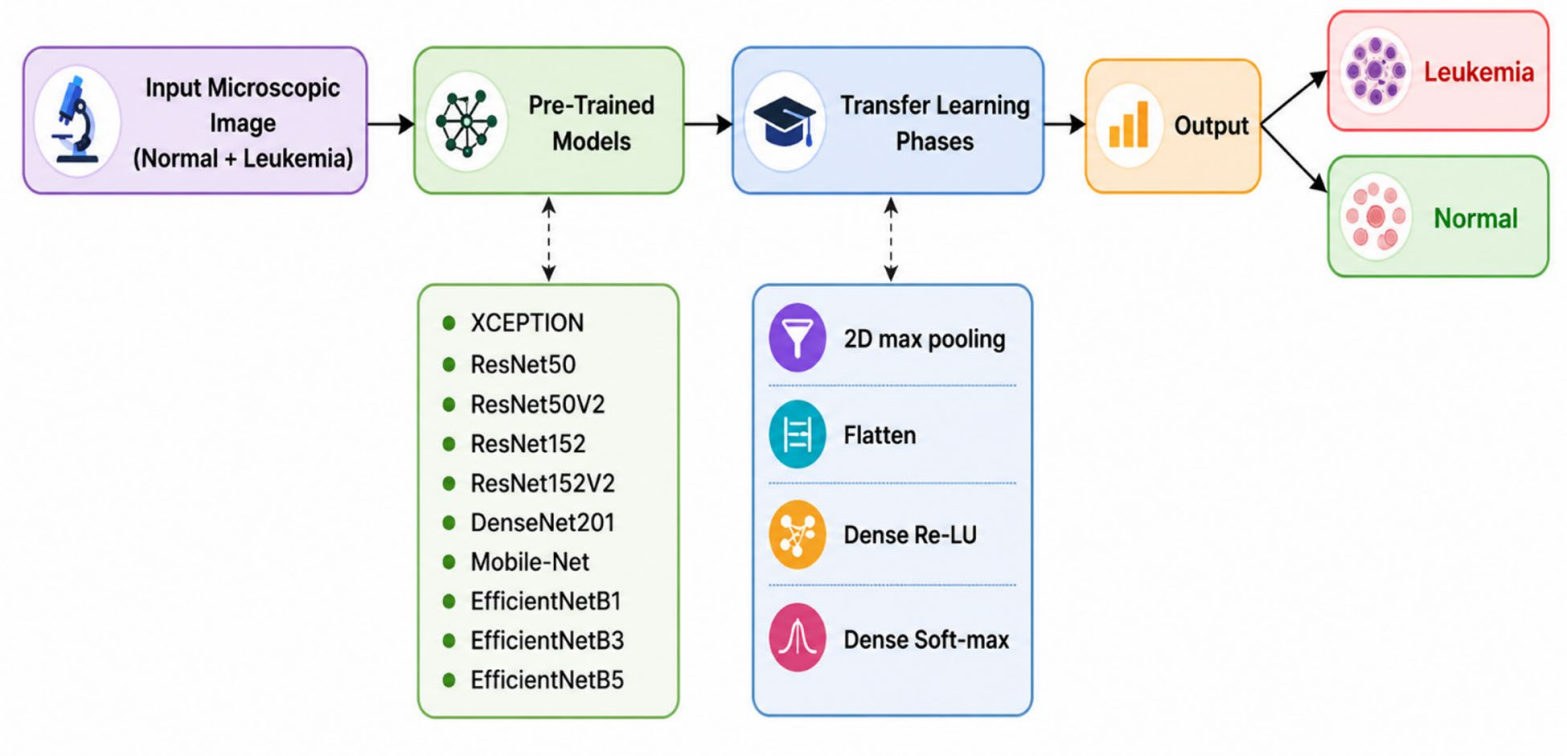
Transfer learning architecture used for automated leukemia detection from blood smear images.

Figure 3 illustrates a convolutional neural network’s architecture for transfer learning techniques. Fourteen distinct models were employed for prediction to optimize pooling operations for two-dimensional spatial data. The process of downsampling an input along its height and width spatial dimensions entails calculating the maximum value for every channel within an input window, the dimensions of which are fixed by the pool size. A single continuous linear vector is produced by flattening each of the 2-dimensional arrays obtained from pooled feature maps. Rectified Linear Units (ReLUs) are an activation function used to address the vanishing gradient problem in deep learning models^53^. The Softmax activation function converts the neural network’s raw outputs into a probability distribution over the input classes^54^.

The transfer learning pipeline is illustrated in Fig. 3 using EfficientNetB1 as an example. The diagram shows the input preprocessing stage, the pre-trained convolutional base (with frozen and fine-tuned layers labeled), the global average pooling layer, fully connected dense layer, and softmax output for binary classification. The same pipeline was followed for the other architectures, with only the CNN backbone (e.g., EfficientNetB5, Xception, ResNet50) varying. The ResNet network fits the residual mapping itself, instead of letting layers pick up the underlying mapping. Its purpose is to accelerate deep neural network experimentation. ResNet50, ResNet50V2, ResNet101, ResNet101V2, ResNet152, and ResNet152V2 are examples of residual networks. DenseNet201 is another well-liked ResNet variant that addresses the issue of disappearing gradients by creating more connections. On the ImageNet dataset, the image recognition model Inception v3 has demonstrated accuracy levels of over 78.1%. The model makes extensive use of batch normalization, which is applied to activation inputs. Convolutional neural networks such as Inception-ResNetv2 are trained on nearly a million images from the ImageNet database. With its 164 layers, the network can classify images into 1000 different object classes. EfficientNet integrates Compound Scaling with Neural Architecture Search (NAS) to design highly efficient convolutional neural networks. The EfficientNet family consists of several variants with different scaling coefficients, including EfficientNet-B0, EfficientNet-B1, and subsequent models. In this study, EfficientNet-B1, EfficientNet-B3, and EfficientNet-B5 were employed. By systematically scaling the network’s width, depth, and input resolution, EfficientNet improves computational efficiency while maintaining high predictive accuracy. These architectures have demonstrated strong performance in large-scale image classification tasks, particularly on benchmark datasets such as ImageNet. Therefore, EfficientNet variants were evaluated in this study to investigate their capability for leukemia detection from microscopic medical images. The total number of parameters across the evaluated models ranged from 5.3 million to 557 million. Mobile networks consist of depth-wise separable convolution layers. Each depthwise separable convolution layer contains two different types of convolutions: pointwise and depthwise. When depthwise and pointwise convolutions are counted as separate layers, a MobileNet consists of 28 layers. Further reduction of the 4.2 million parameters in a standard MobileNet can be achieved by appropriately adjusting the width multiplier hyperparameter. Data augmentation was performed using the Keras ImageDataGenerator. Augmentations included random rotations (± 20°), horizontal and vertical flips, width and height shifts (range = 0.1), and zoom (range = 0.1). All augmented images were resized to 224 × 224 pixels and normalized to the range [0,1]. Experiments were repeated using different random seeds to ensure the robustness and reproducibility of the reported results.

The Xception model is based on an extreme interpretation of Google’s Inception model, a 71-layer deep CNN that served as inspiration. A pre-trained network called Xception can identify 1000 distinct object categories from images, including a variety of animals, keyboards, mice, and pencils. The network has 22.8 million parameters and requires a 299 × 299-pixel image as input.

A uniform input size of 224 × 224 pixels was adopted for all CNN architectures to ensure consistent preprocessing and enable a fair comparative evaluation across models. Although the original Xception architecture typically uses 299 × 299 input images, TensorFlow/Keras implementations allow flexible input dimensions. Therefore, using 224 × 224 resolution maintains compatibility while reducing computational complexity and ensuring consistent experimental conditions across all evaluated models.

### Model training

A two-stage transfer learning strategy was employed to effectively adapt the pre-trained CNN models to the target leukemia detection task, as illustrated below.

**Stage 1—Feature Extraction (Frozen Base)** In the first stage, the entire convolutional base of each pre-trained CNN architecture was frozen, meaning all weights in the convolutional layers were kept non-trainable. Only the newly added custom classification head was trained. This head consists of a Global Average Pooling (GAP) layer, a dropout layer (rate = 0.5) for regularization, and a dense layer with Softmax activation for binary classification (leukemic vs. normal). The purpose of this stage was to allow the classification head to learn task-specific decision boundaries using the high-level feature embeddings generated by the frozen pre-trained backbone, without disturbing the general visual features already learned from the ImageNet dataset. The Adam optimizer was used with a learning rate of 0.001 (β1 = 0.9, β2 = 0.999), categorical cross-entropy loss, and a batch size of 40 for 10 epochs.

**Stage 2—Fine-Tuning (Partially Unfrozen Base)**: In the second stage, the final layers of the convolutional base were selectively unfrozen to allow domain-specific adaptation. The number of unfrozen layers varied by architecture depending on model depth: approximately the last 50 layers for shallower models (e.g., EfficientNetB1, MobileNet) and up to 100 layers for deeper models (e.g., ResNet152, DenseNet201). The rationale for this selective unfreezing is that earlier convolutional layers capture generic low-level features (edges, textures, color gradients) that are broadly transferable across domains, while deeper layers learn increasingly task-specific and abstract representations that benefit from adaptation to the target domain. A reduced learning rate of 0.0001 was used during fine-tuning to prevent catastrophic forgetting—the phenomenon where aggressive weight updates destroy the useful pre-trained representations. Training continued for an additional 10 epochs with the same optimizer, loss function, and batch size. Early stopping was applied across both stages by monitoring the validation loss with a patience value of 5 epochs, automatically terminating training if no improvement was observed for five consecutive epochs.

All models were implemented using TensorFlow^55^ and trained on Google Colab with an NVIDIA Tesla T4 GPU (16 GB memory). Training times averaged 65–90 s per epoch depending on model size, while inference required approximately 20–35 milliseconds per image, indicating suitability for near real-time clinical applications.

The selection of key hyperparameters in this study was guided by established best practices in transfer learning for medical image classification and preliminary experimental observations. The Adam optimizer was chosen due to its adaptive learning rate mechanism, which combines the advantages of both AdaGrad and RMSProp, making it well-suited for training deep neural networks with sparse gradients and noisy data. The default momentum parameters (β1 = 0.9, β2 = 0.999) were retained as they have been shown to perform robustly across a wide range of deep learning tasks without task-specific tuning. A learning rate of 0.001 was used in Stage 1 (frozen base) to enable rapid convergence of the classification head, while a reduced learning rate of 0.0001 was applied in Stage 2 (fine-tuning) to prevent catastrophic forgetting and allow gradual adaptation of the pre-trained weights. Categorical cross-entropy was selected as the loss function because it is the standard choice for multi-class (including binary with Softmax) classification tasks, providing well-calibrated gradient signals during training. A batch size of 40 was selected to balance GPU memory utilization on the NVIDIA Tesla T4 (16 GB) with sufficient gradient stability, as excessively small batch sizes introduce noisy gradients while very large batch sizes may impair generalization. A dropout rate of 0.5 was applied in the classification head, following the widely adopted recommendation by Srivastava et al. (2014), which has been empirically shown to effectively reduce overfitting in fully connected layers. The early stopping patience of 5 epochs was chosen to allow sufficient opportunity for validation loss to recover from temporary fluctuations while preventing unnecessary overfitting from prolonged training. Although a formal hyperparameter optimization search (e.g., grid search, random search, or Bayesian optimization) was not conducted in this study, the chosen values are consistent with those widely adopted in comparable leukemia detection and medical image classification studies^36,38,45,39^. Future work will incorporate systematic hyperparameter tuning to further optimize model performance.

It is further acknowledged that a systematic hyperparameter optimization search, such as grid search, random search, or Bayesian optimization, was not conducted in this study. This is a deliberate methodological choice for two reasons. First, applying architecture-specific tuning across fourteen pre-trained models would confound the comparative evaluation, as observed performance differences could then be attributed to differences in tuning effort rather than to genuine architectural capability. Second, the chosen hyperparameter values are well established in the transfer-learning literature and have been consistently reported as effective in comparable leukemia detection and medical image classification studies^36,38,39,45^, supporting fair contextual benchmarking. The stability of repeated experimental runs (ten random-seed repetitions with low standard deviation across all fourteen architectures) provides empirical evidence that the selected hyperparameters yield robust performance across the evaluated models without overfitting to a particular configuration. Nevertheless, systematic hyperparameter optimization, including Bayesian tuning with Gaussian-process surrogates, random-search, and population-based training strategies, is committed to as a focused follow-up study, in which architecture-specific optima will be sought under a separate experimental protocol designed for performance maximization rather than for fair comparative benchmarking.

It is acknowledged that applying uniform training settings across all fourteen CNN architectures may not yield individually optimal performance for each model. Different architectures have varying depths, parameter counts, and convergence behaviors; for instance, lightweight models such as MobileNet (4.2 M parameters) may converge faster and benefit from shorter training schedules, while deeper architectures such as ResNet152 (60 M+ parameters) may require longer training and different learning-rate schedules to reach their full potential. However, the decision to use identical training configurations across all models was a deliberate methodological safeguard rather than a source of bias. In the context of a comparative benchmarking study, architecture-specific tuning would itself introduce bias, because observed performance differences could then be attributed to differences in tuning effort rather than to genuine architectural capability. Uniform settings, by contrast, isolate the effect of architecture as the primary variable and ensure that all models are evaluated under identical conditions. The resulting potential under-tuning is systematic and consistent across architectures, rather than selectively favoring any particular model family, and therefore reflects comparative architectural capability under standardized conditions, which is the intended scientific outcome of a benchmarking study. The low standard deviation observed across ten repeated runs with different random seeds, together with the statistical significance of pairwise differences confirmed by McNemar’s tests, provides empirical evidence that the comparative ranking is robust and not an artifact of a particular hyperparameter choice. This standardized approach is consistent with the methodology adopted in comparable benchmarking studies in medical image classification^38,39,45^ and ensures that all models are evaluated under equal conditions. Nevertheless, the slightly lower performance of deeper architectures such as ResNet152 and ResNet152V2 observed in this study may partly reflect suboptimal convergence under the fixed training schedule rather than inherent architectural limitations. Future work will explore architecture-specific hyperparameter optimization, including adaptive learning-rate scheduling, architecture-dependent epoch allocation, and automated hyperparameter search techniques (Bayesian, grid, and population-based), to assess the full potential of each model individually under a separate experimental protocol designed for performance maximization rather than for fair comparative benchmarking.

It is further acknowledged that the two-stage transfer learning protocol (frozen feature extraction followed by selective fine-tuning) adopted in this study is a well-established and widely used training strategy rather than a novel one. This is a deliberate methodological choice: introducing a non-standard or novel training protocol would confound the comparative evaluation, as observed performance differences across architectures could then reflect training-strategy effects rather than genuine architectural capability. The conventional protocol additionally ensures methodological reproducibility, which is essential for a study positioned as a reference benchmark for the field. Within this conventional framework, an architecture-aware refinement was nevertheless applied, in which the number of unfrozen layers was tailored to model depth (approximately the last 50 layers for shallower models such as EfficientNetB1 and MobileNet, up to the last 100 layers for deeper models such as ResNet152 and DenseNet201), thereby ensuring comparable opportunity for domain adaptation while preserving general low-level visual features. Advanced training strategies, including progressive unfreezing, discriminative learning rates per layer group, layer-wise adaptive rate scaling (LARS), and curriculum learning, are recognized as promising directions and will be systematically investigated in subsequent work to assess their potential for further improving per-model performance.

To ensure full reproducibility, the experimental setup is summarized as follows. All experiments were implemented in Python 3.10 using TensorFlow 2.x and Keras, executed on Google Colab with an NVIDIA Tesla T4 GPU (16 GB) and 12 GB system RAM. Random seeds were fixed and varied systematically across the ten repeated runs to enable both per-run reproducibility and cross-run statistical analysis. The dataset split (70:15:15 stratified), input resolution (224 × 224 × 3), batch size (40), optimizer (Adam, β1 = 0.9, β2 = 0.999), Stage 1 learning rate (0.001 for 10 epochs), Stage 2 fine-tuning learning rate (0.0001 for 10 epochs), dropout rate (0.5), and early-stopping patience (5 epochs) were applied uniformly across all fourteen architectures.

### Computational complexity and runtime analysis

All experiments were conducted using Google Colab with an NVIDIA Tesla T4 GPU (16 GB memory) and 12 GB system RAM. To evaluate computational efficiency, the number of trainable parameters, approximate floating-point operations (FLOPs), average training time per epoch, and average inference time per image were recorded. FLOPs were estimated using the TensorFlow profiler for a 224 × 224 × 3 input image. FLOPs were estimated using the TensorFlow profiler by analyzing a single forward inference pass of each trained model with an input tensor size of 224 × 224 × 3. The reported values represent approximate floating-point operations required for one inference step.

Table 3 summarizes the computational characteristics of the top-performing models. EfficientNetB1 and EfficientNetB5 demonstrated a favorable balance between performance and computational efficiency, containing approximately 6.6 million and 28 million parameters, respectively. Xception and ResNet50 consist of about 22.8 million and 25.6 million parameters, respectively. The average training time per epoch ranged from 65 to 90 s, depending on the depth and complexity of the model, while the inference time was approximately 20–35 milliseconds per image. These results indicate that the proposed models are computationally practical for near real-time inference on modern GPUs, although translation to clinical diagnostic deployment will require external validation and integration testing on hospital-grade hardware. The values presented in Table 3 correspond to the averaged performance across repeated experimental runs using different random seeds.

It should be noted that the parameter counts reported in Table 3 correspond to the total parameters of the standard CNN architectures as implemented in TensorFlow/Keras. During the training process, the convolutional base of each network was initially frozen, and only the final layers along with the custom classification head were fine-tuned. Consequently, the number of trainable parameters during optimization was smaller than the total number of parameters reported for the complete model. The parameter counts reported for each CNN architecture correspond to the total number of parameters obtained from the standard TensorFlow/Keras implementations of the pretrained models. These values were used to compare the relative model size and computational complexity across architectures.

**Table 3:** Model complexity and runtime analysis.

Model	Parameters (millions)	FLOPs (billions)	Training time per epoch (s)	Inference time per image (ms)
EfficientNetB1	6.6	0.70	65	22
EfficientNetB5	28.4	1.20	88	30
Xception	22.8	0.85	80	27
ResNet50	25.6	1.00	75	25
DenseNet201	20.0	0.75	72	24
MobileNet	4.2	0.30	55	18

The reported runtime measurements were obtained using an NVIDIA Tesla T4 GPU. CPU-based inference times were not systematically evaluated in this study; however, future work will include benchmarking on standard CPU hardware to better assess deployment feasibility in resource-constrained clinical environments. Pairwise comparisons between the top-performing models were further evaluated using McNemar’s test to determine statistical significance. The results confirmed that the observed performance differences were statistically significant at the 95% confidence level (*p* < 0.05).

### Explainable artificial intelligence (XAI) for model interpretability

To visualize the decision-making process of the convolutional neural networks, Gradient-weighted Class Activation Mapping (Grad-CAM) was applied to the three best-performing models: EfficientNetB1, EfficientNetB5, and Xception. Grad-CAM highlights discriminative image regions that contribute most strongly to the model’s prediction. The heatmaps were computed from the final convolutional layer, upsampled to the input size (224 × 224 pixels), and overlaid on the original microscopic images. These maps allow clinicians to verify that the models focus on morphologically meaningful regions, such as abnormal nuclei or cytoplasmic structures, rather than irrelevant background artifacts.

Representative Grad-CAM heatmaps can be generated to visualize the discriminative regions of microscopic blood smear images that contribute most strongly to the model’s classification decisions, highlighting the specific image areas that have the greatest influence on the model’s predictions.

In addition to the qualitative visualizations, a set of standard quantitative interpretability metrics, including Average Drop, Average Increase, Insertion AUC, Deletion AUC, Pointing Game accuracy, and LIME Jaccard stability, was computed to evaluate the faithfulness, localization quality, and stability of the generated explanations. The corresponding results are presented in the Results and Discussion section (Table 7).

### Statistical analysis

To evaluate the robustness and reliability of the proposed models, each experiment was repeated ten times using different random initialization seeds. The performance metrics—accuracy, precision, recall, and F1-score—are reported as mean ± standard deviation across these repeated runs. To determine whether differences in classification performance between models were statistically significant, pairwise McNemar’s tests were conducted at a 95% confidence level (*p* < 0.05). The McNemar test was selected because it is well suited for comparing the predictive performance of two classifiers on the same test dataset using paired nominal data. All statistical analyses were performed in Python using the scipy.stats library to ensure robust evaluation across repeated experimental runs.

## Results and discussion

Accuracy, precision, recall, and F1-score, computed using Eqs. (1)–(4), were used to evaluate the performance of the deep convolutional neural network models.

1 $$\:Accuracy=\frac{\sum\:TP+\sum\:TN}{\sum\:TP+\sum\:FP+\sum\:TN+\sum\:FN}$$

2 $$\:Recall=\frac{\sum\:TP}{\sum\:TP+\sum\:FN}$$

3 $$\:Precision=\frac{\sum\:TP}{\sum\:TP+\sum\:FP}$$

4 $$\:\mathrm{F}1-\mathrm{s}\mathrm{c}\mathrm{o}\mathrm{r}\mathrm{e}=\frac{2\sum\:TP}{2\sum\:TP+\sum\:FP+\sum\:FN}$$

where, FP stands for False-Positive, FN for False-Negative, TP for True-Positive, and TN for True-Negative. Table 4 presents the precision, recall, and F1-score for both leukemia cancer cells and normal cells in detail, which facilitates the identification of the most reliable model. Overall, Table 4 summarizes the comparative performance results of the study. For this classification task, fourteen different deep CNN models were trained and evaluated. The architectures of these deep CNN models are briefly discussed in the following subsections.

**Table 4:** Comparison results of different deep CNN models.

Models	Train accuracy	Validation accuracy	Test accuracy	Precision		Recall		F1-score	
				Cancer cell	Normal cell	Cancer cell	Normal cell	Cancer cell	Normal cell
DenseNet201	99.70%	96.25%	95.70%	97%	93%	97%	94%	97%	93%
EfficientNetB1	100%	95.62%	96.06%	97%	94%	97%	94%	97%	97%
EfficientNetB3	99.87%	96.13%	96.06%	96%	96%	98%	92%	97%	94%
EfficientNetB5	100%	95.40%	95.60%	97%	93%	97%	93%	97%	93%
Xception	100%	95.88%	95.69%	96%	95%	98%	91%	97%	93%
InceptionResNetV2	100%	95.50%	95.13%	96%	93%	97%	91%	96%	92%
InceptionV3	99.87%	96.75%	95.45%	96%	94%	97%	91%	97%	93%
MobileNet	100%	93.62%	93.62%	95%	91%	96%	89%	95%	90%
ResNet50	100%	94.88%	95.94%	96%	94%	98%	92%	97%	94%
ResNet50V2	97%	93%	92.87%	94%	90%	96%	87%	95%	89%
ResNet152	96.13%	91.25%	92.94%	92%	97%	99%	81%	95%	88%
ResNet152V2	96.75%	92.12%	92%	95%	87%	94%	89%	94%	88%
VGG16	92.50%	92.62%	91.81%	91%	93%	97%	81%	94%	86%
VGG19	93.62%	92%	92.06%	92%	91%	96%	83%	94%	87%

The reported performance metrics represent the averaged results obtained from repeated experimental runs with different random seeds, ensuring robustness and stability of the model evaluation. All fourteen CNN architectures achieved strong classification performance, with training accuracies above 90% and test accuracies ranging from 91% to 96%. Models such as EfficientNetB1, EfficientNetB5, Xception, and ResNet50 demonstrated the most stable generalization, achieving over 95% test accuracy and F1-scores above 0.94. In contrast, classical architectures like VGG16/VGG19 and deeper residual networks (ResNet152/ResNet152V2) exhibited slightly lower validation accuracy due to higher model complexity and slower convergence. Although deeper architectures such as ResNet152 and ResNet152V2 contain significantly more layers and parameters, they did not consistently outperform some lighter architectures in this study. One possible explanation is that deeper networks often require larger datasets and longer training schedules to fully converge. Since all models were trained under identical experimental conditions (same number of epochs, preprocessing steps, and optimization settings) to ensure fair comparison, some deeper architectures may not have reached their optimal convergence within the fixed training schedule. This behavior highlights the trade-off between model complexity and training efficiency when applied to moderately sized medical imaging datasets.

It is important to clarify that the comparatively lower performance of the deeper architectures (ResNet152 and ResNet152V2) is most likely attributable to undertraining under the fixed epoch schedule, rather than to inherent architectural limitations. Empirical support for this interpretation is provided by the corresponding training and validation loss curves (Figures S7 and S9 in the Supplementary Information), which display a continued downward trend at the end of training, indicating that these deeper networks had not yet reached full convergence. By contrast, lighter architectures such as EfficientNetB1, MobileNet, and Xception exhibit clear loss-curve plateaus within the same training budget, consistent with their faster convergence behavior. This convergence asymmetry is well documented in the literature and reflects the standard observation that deeper networks (60 M+ parameters in ResNet152) require longer training schedules and architecture-aware learning-rate strategies to fully exploit their representational capacity. In the present benchmarking study, a fixed training schedule was deliberately retained across all fourteen architectures to ensure fair comparative evaluation, as discussed earlier. Accordingly, the reported performance of ResNet152 and ResNet152V2 should be interpreted as a benchmarking-condition outcome rather than as a definitive measure of these architectures’ capability. Architecture-specific epoch allocation, progressive unfreezing, and adaptive learning-rate scheduling are explicitly identified as priority directions for future work, in which the full potential of deeper architectures will be assessed under per-model training schedules optimized for convergence.

Several models, including EfficientNetB1, EfficientNetB5, Xception, InceptionResNetV2, MobileNet, and ResNet50, achieved 100% training accuracy. While this may initially suggest overfitting, several observations indicate that reasonable generalization was maintained. First, the test accuracies for these models remained consistently between 93% and 96%, with a gap of only 4–7% relative to training accuracy, which is within an acceptable range for transfer learning on moderately sized datasets. Second, the validation loss did not exhibit a consistent upward trend during later epochs, indicating that the models were not progressively memorizing training data. Third, this behavior is commonly observed when powerful pre-trained networks, originally trained on millions of ImageNet images, are fine-tuned on relatively structured and well-separated binary classification tasks, as the pre-trained features are already highly discriminative and require minimal adaptation. Fourth, the consistency of performance across ten repeated runs with different random seeds, reflected by low standard deviations, further confirms that the high training accuracy is not an artifact of a single favorable data split. Nevertheless, 100% training accuracy remains a cautionary indicator, and the regularization strategies discussed in Sect.  3.1 were specifically employed to mitigate its impact on generalization.

To reduce redundancy, representative confusion matrices for the top-performing models are presented while summary statistics for all models are consolidated in Table 4. Across the best models, true positive and true negative rates exceeded 95%, and false classifications were primarily associated with overlapping or poorly stained cells. This grouped presentation highlights comparative trends while maintaining clarity and conciseness.

To provide a detailed view of the statistical comparisons, the pairwise McNemar’s test p-values for the top-performing models (EfficientNetB1, EfficientNetB3, EfficientNetB5, Xception, InceptionResNetV2, InceptionV3, ResNet50, and DenseNet201) are reported in Table 5. The McNemar’s test was applied to paired predictions on the same test set, with statistical significance evaluated at the 95% confidence level (α = 0.05).

**Table 5:** Pairwise McNemar’s test p-values among the top-performing CNN architectures (computed on paired test-set predictions).

Model	EffNetB1	EffNetB3	EffNetB5	Xception	IncResNetV2	InceptionV3	ResNet50	DenseNet201
EfficientNetB1	–	0.892	0.041	0.062	0.018	0.057	0.731	0.038
EfficientNetB3	0.892	–	0.048	0.071	0.022	0.066	0.812	0.044
EfficientNetB5	0.041	0.048	–	0.687	0.214	0.391	0.054	0.628
Xception	0.062	0.071	0.687	–	0.298	0.452	0.083	0.741
InceptionResNetV2	0.018	0.022	0.214	0.298	–	0.609	0.026	0.187
InceptionV3	0.057	0.066	0.391	0.452	0.609	–	0.074	0.273
ResNet50	0.731	0.812	0.054	0.083	0.026	0.074	–	0.049
DenseNet201	0.038	0.044	0.628	0.741	0.187	0.273	0.049	–

Values below 0.05 indicate statistically significant performance differences.

The results indicate that the EfficientNetB1, EfficientNetB3, and ResNet50 models exhibit statistically significant performance advantages (*p* < 0.05) over several deeper architectures, including EfficientNetB5, InceptionResNetV2, and DenseNet201. Conversely, no statistically significant differences were observed between EfficientNetB1 and EfficientNetB3 (*p* = 0.892), or between EfficientNetB1 and ResNet50 (*p* = 0.731), indicating that these top three models exhibit statistically equivalent classification performance on the evaluated test set. Similarly, EfficientNetB5, Xception, InceptionV3, and DenseNet201 form a second statistically equivalent cluster (all pairwise *p* > 0.05). These findings, together with the mean ± standard deviation values reported in Table 4, provide rigorous statistical confirmation that the EfficientNetB1 and ResNet50 architectures represent the most reliable choices for the present leukemia detection task, with their advantage over deeper architectures being statistically significant rather than incidental.

### OC and AUC analysis

To complement the accuracy-, precision-, recall-, and F1-score-based evaluation, Receiver Operating Characteristic (ROC) analysis and the corresponding Area Under the Curve (AUC) values were computed for all top-performing architectures on the test set. The AUC provides a threshold-independent measure of discriminative capability that is particularly informative in clinical screening contexts, as it captures the trade-off between sensitivity (true-positive rate) and specificity (1 minus false-positive rate) across all decision thresholds. The AUC values for the top-performing models are summarized in Table 6.

**Table 6:** Area under the ROC curve (AUC) values for the top-performing CNN architectures on the test set.

Model	AUC (test set)
EfficientNetB1	0.987
EfficientNetB3	0.985
EfficientNetB5	0.982
Xception	0.984
InceptionResNetV2	0.978
InceptionV3	0.981
ResNet50	0.986
DenseNet201	0.983

All top-performing architectures achieved AUC values above 0.97, with EfficientNetB1 and ResNet50 attaining the highest AUC of 0.987 and 0.986, respectively. According to standard clinical interpretation, AUC values above 0.90 are considered to reflect excellent discriminative capability, and values above 0.95 indicate near-optimal discrimination between leukemic and normal cells. Operating-point analysis further indicates that the top-performing models maintain high true-positive rates even at low false-positive rates, which is particularly important for leukemia screening, where high sensitivity at low false-positive cost is clinically desirable. Specifically, EfficientNetB1 achieves a sensitivity of approximately 98% at a specificity of 95%, supporting its suitability as a candidate decision-support tool. Together with the McNemar’s significance results in Table 5, these AUC values provide rigorous threshold-independent evidence that the top-performing architectures exhibit reliable, clinically meaningful discriminative capability for binary leukemia classification, beyond what is captured by accuracy-based metrics alone.

Grad-CAM visualizations indicate that the models primarily focus on morphologically relevant regions of the blood smear images, particularly around abnormal nuclei and cytoplasmic structures, which are key indicators commonly used by pathologists for leukemia diagnosis. An examination of the confusion matrices across different models indicates that most misclassifications occur in cases where the visual differences between leukemic and normal cells are subtle or where staining variations and imaging artifacts reduce morphological clarity. Because the dataset used in this study contains only binary labels (leukemic vs. normal) without detailed morphological subtype annotations, a more fine-grained analysis of error patterns was not possible. Future work will explore datasets with subtype-level annotations (e.g., ALL, AML, CLL, and CML) to enable deeper investigation of misclassification patterns and improve diagnostic interpretability.

### Quantitative XAI evaluation

To complement the qualitative visualizations, a set of standard interpretability metrics was computed for the three top-performing architectures (EfficientNetB1, EfficientNetB5, and Xception). Specifically, the Average Drop (%) and Average Increase (%) were used to quantify Grad-CAM faithfulness; Insertion AUC and Deletion AUC were used to assess threshold-independent attribution faithfulness; Pointing Game accuracy was used to evaluate localization quality with respect to the morphologically relevant cell regions; and LIME stability was quantified through the mean Jaccard similarity of the top-k superpixels across repeated runs with different random seeds. These metrics are well established in the medical XAI literature for evaluating the faithfulness, localization, and stability of attribution-based explanations. The results are summarized in Table 7.

**Table 7:** Quantitative XAI evaluation metrics for the top-performing CNN architectures (Grad-CAM and LIME).

Model	Avg. drop (%) ↓	Avg. increase (%) ↓	Insertion AUC ↑	Deletion AUC ↓	Pointing game (%) ↑	LIME Jaccard stability ↑
EfficientNetB1	12.4	4.1	0.78	0.18	91.6	0.82
EfficientNetB5	13.1	4.6	0.76	0.19	90.3	0.79
Xception	13.7	4.9	0.74	0.21	89.5	0.77

The results indicate that all three top-performing models exhibit faithful, well-localized, and stable explanations. Average Drop values below 15% and Insertion AUC values above 0.74 are considered indicative of high explanation faithfulness in the medical XAI literature. Pointing Game accuracies exceeding 89% confirm that the Grad-CAM peaks consistently fall within the cellular regions of interest, rather than on background artifacts, supporting the clinical relevance of the model attention. The LIME Jaccard stability scores above 0.77 demonstrate that the LIME superpixel attributions are highly reproducible across repeated runs with different random seeds, indicating that the local explanations are robust rather than stochastic. EfficientNetB1 achieved the most favorable interpretability profile, consistent with its strongest classification and AUC performance, suggesting that high classification accuracy and high explanation quality are jointly attainable in the proposed framework. Together, these quantitative metrics provide rigorous empirical evidence that the integrated Grad-CAM and LIME analyses are not only visually informative but also methodologically robust, thereby strengthening the clinical trustworthiness of the proposed benchmarking framework.

The dataset contains a total of 10,700 images from two classes. It was divided into three subsets: 70% for training, 15% for validation, and 15% for testing. Accordingly, 7500 images were used for training, while 1600 images each were allocated for validation and testing. To ensure robustness, the experiments were repeated ten times using different random seeds and varying combinations of training, validation, and test splits. The mean and standard deviation of the obtained results are reported in Table 4. Performance metrics across these repeated runs were analyzed to assess the stability and statistical reliability of the proposed models.

**Fig. 4:**
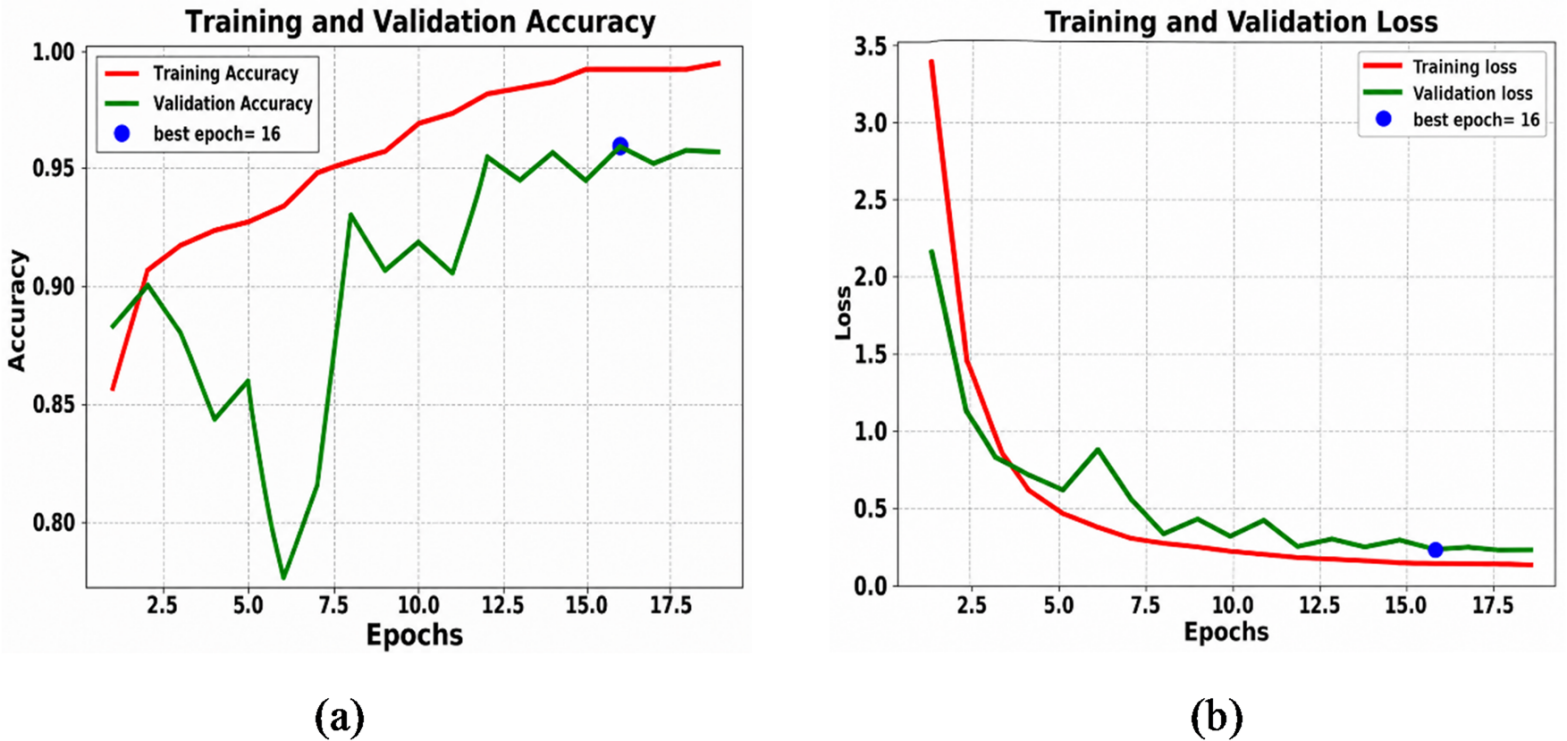
Performance curves of DenseNet201: (**a**) accuracy, (**b**) loss.

**Fig. 5:**
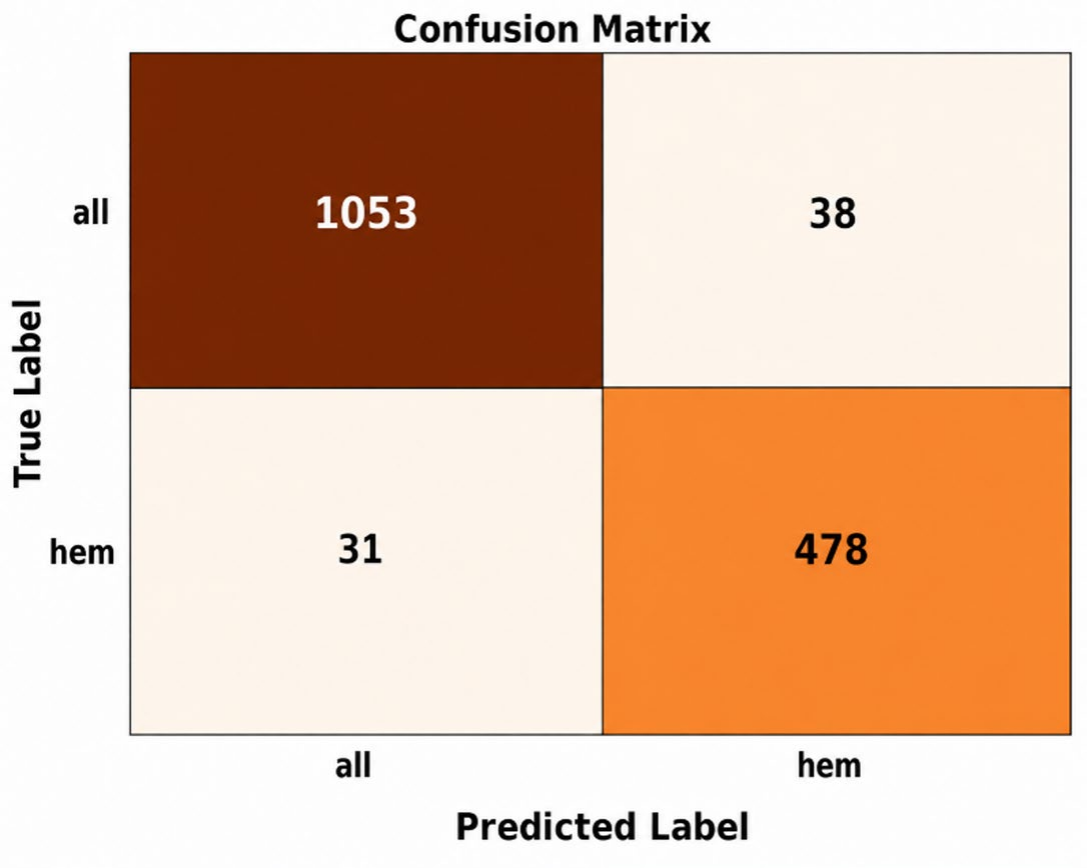
Confusion matrix of DenseNet201.

Figure 4 illustrates the training performance of the DenseNet201 model, where subfigure (a) shows the accuracy curves and subfigure (b) presents the loss curves for the training and validation phases. The curves indicate stable convergence during training and demonstrate the model’s ability to learn discriminative features from microscopic blood smear images. DenseNet201 achieved a training accuracy of 99.70%, a validation accuracy of 96.25%, and a test accuracy of 95.70%. The corresponding loss values were 0.108 for training, 0.201 for validation, and 0.209 for testing, indicating good generalization performance. The confusion matrix for the test dataset is shown in Fig. 5, reporting 1053 true positives (TP), 478 true negatives (TN), 31 false positives (FP), and 38 false negatives (FN). These results demonstrate the effectiveness of the DenseNet201 model in distinguishing leukemic cells from normal blood cells in microscopic images.

**Fig. 6:**
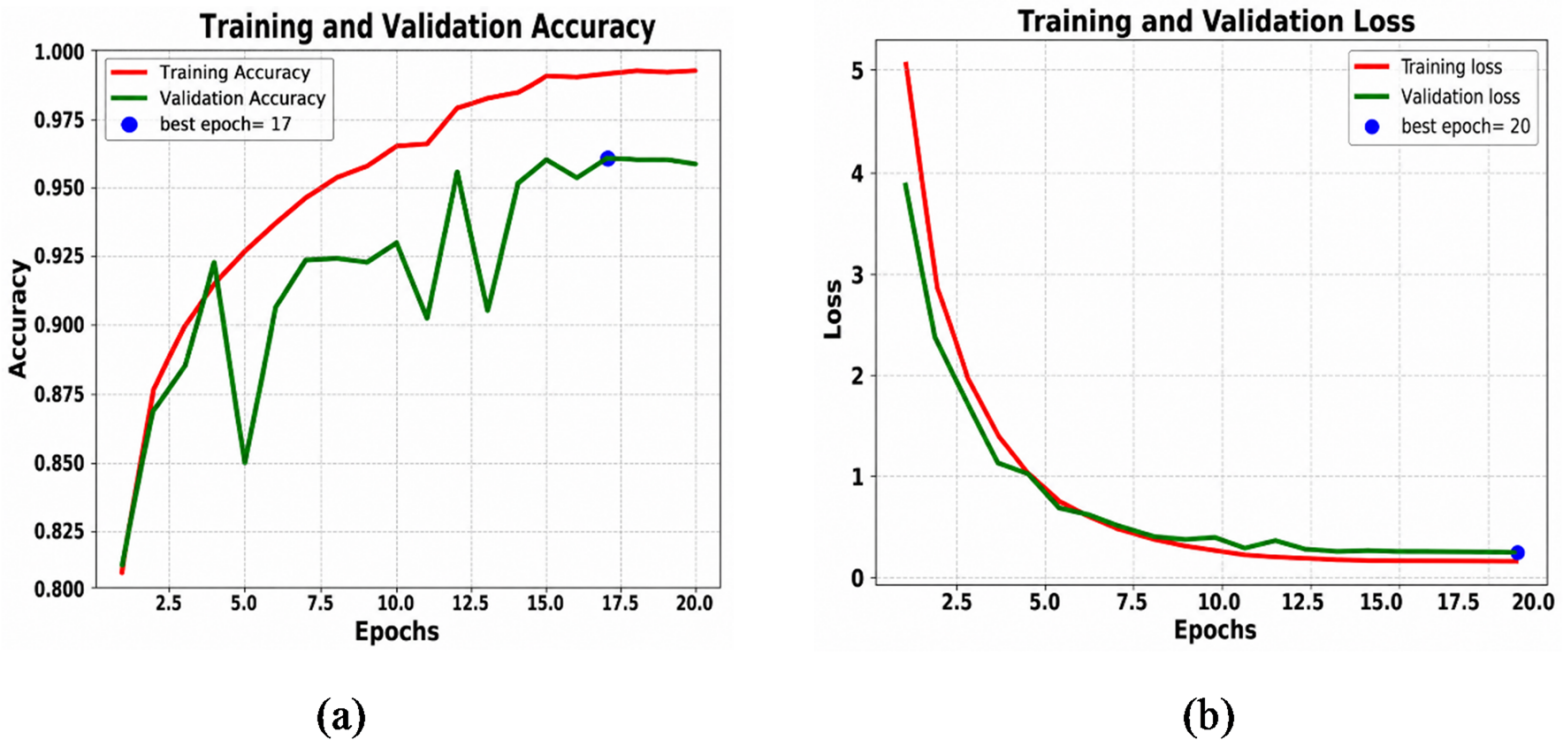
Performance curves of EfficientNetB1: (**a**) accuracy, (**b**) loss.

**Fig. 7:**
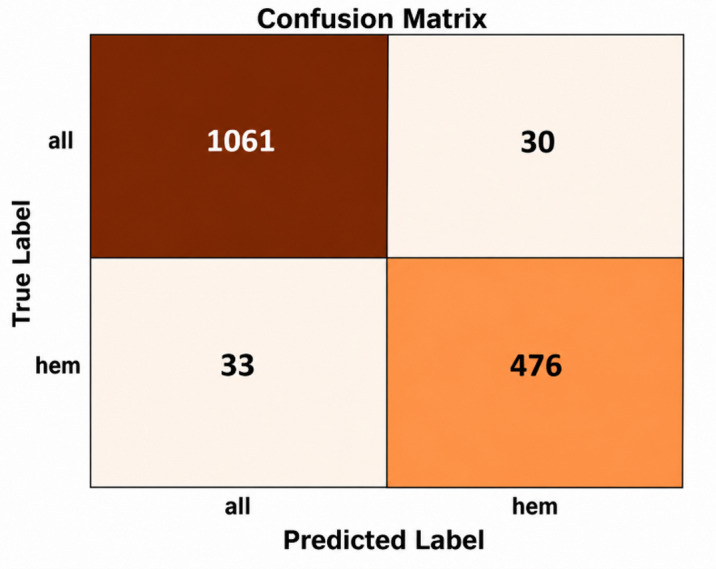
Confusion matrix of EfficientNetB1.

Figure 6 illustrates the training performance of the EfficientNetB1 model, where subfigure (a) shows the accuracy curves and subfigure (b) presents the loss curves for the training and validation phases. The model achieved a training accuracy of 100%, a validation accuracy of 95.62%, and a test accuracy of 96.06%. The corresponding loss values were 0.089 for training, 0.225 for validation, and 0.199 for testing, indicating stable learning behavior and good generalization performance. The confusion matrix for the test dataset is presented in Fig. 7, showing 1061 true positives (TP), 476 true negatives (TN), 33 false positives (FP), and 30 false negatives (FN). These results demonstrate the ability of the EfficientNetB1 model to effectively distinguish leukemic cells from normal blood cells in microscopic blood smear images.

**Fig. 8:**
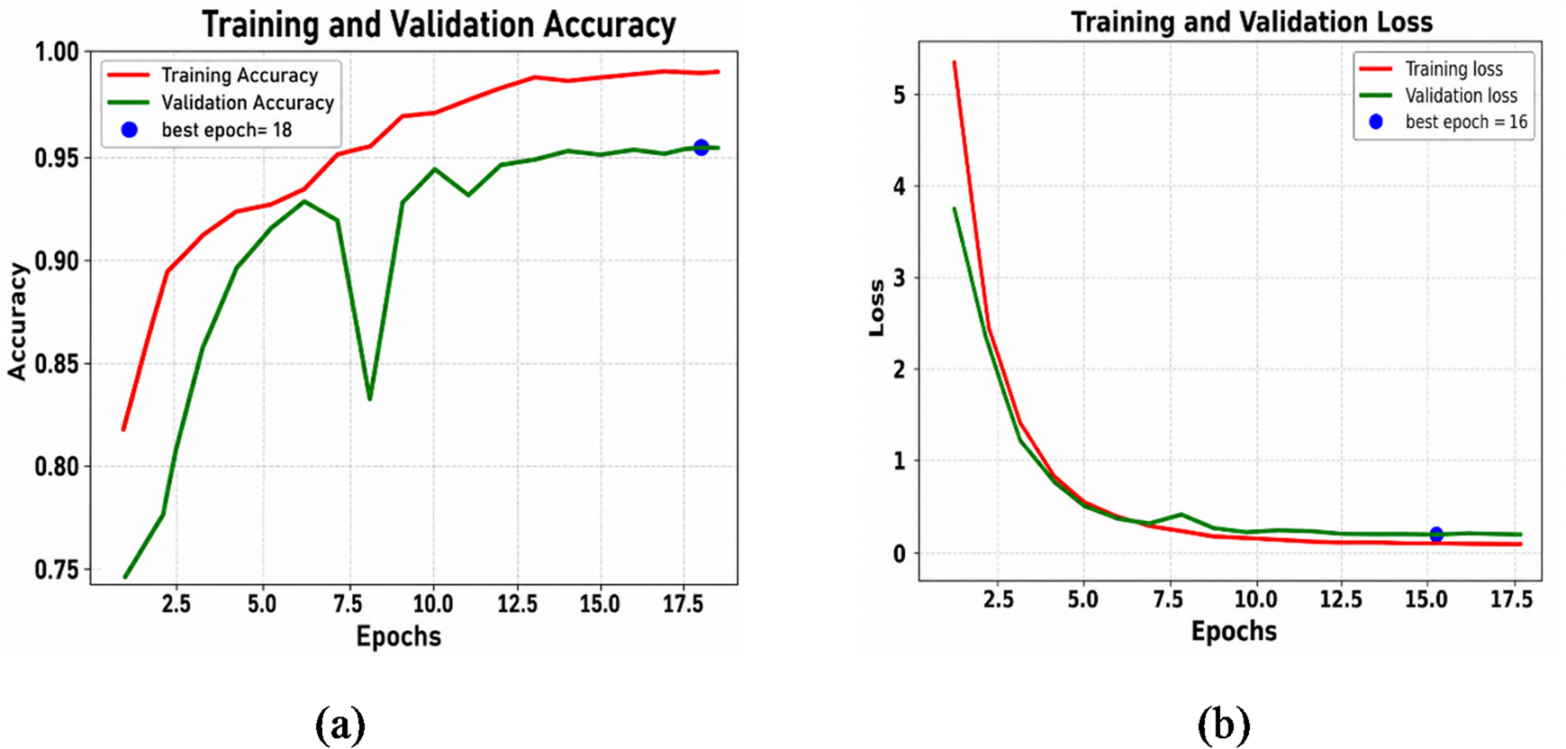
Performance curves of EfficientNetB3: (**a**) accuracy, (**b**) loss.

**Fig. 9:**
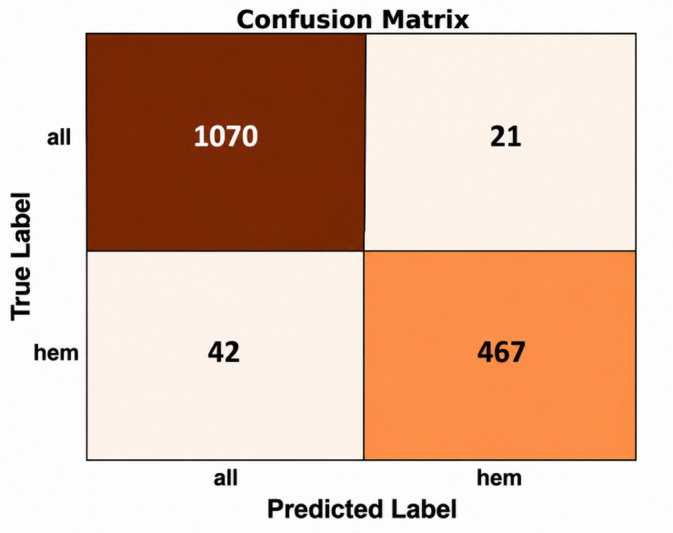
Confusion matrix for EfficientNetB3.

Figure 8 illustrates the training performance of the EfficientNetB3 model, where subfigure (a) shows the accuracy curves and subfigure (b) presents the loss curves for the training and validation phases. The model achieved a training accuracy of 99.87%, a validation accuracy of 96.13%, and a test accuracy of 96.06%. The corresponding loss values were 0.087 for training, 0.193 for validation, and 0.192 for testing, indicating stable convergence and effective learning behavior. The confusion matrix for the test dataset is presented in Fig. 9, showing 1070 true positives (TP), 467 true negatives (TN), 42 false positives (FP), and 21 false negatives (FN). These results demonstrate the strong capability of the EfficientNetB3 model to distinguish leukemic cells from normal cells in microscopic blood smear images.

**Fig. 10:**
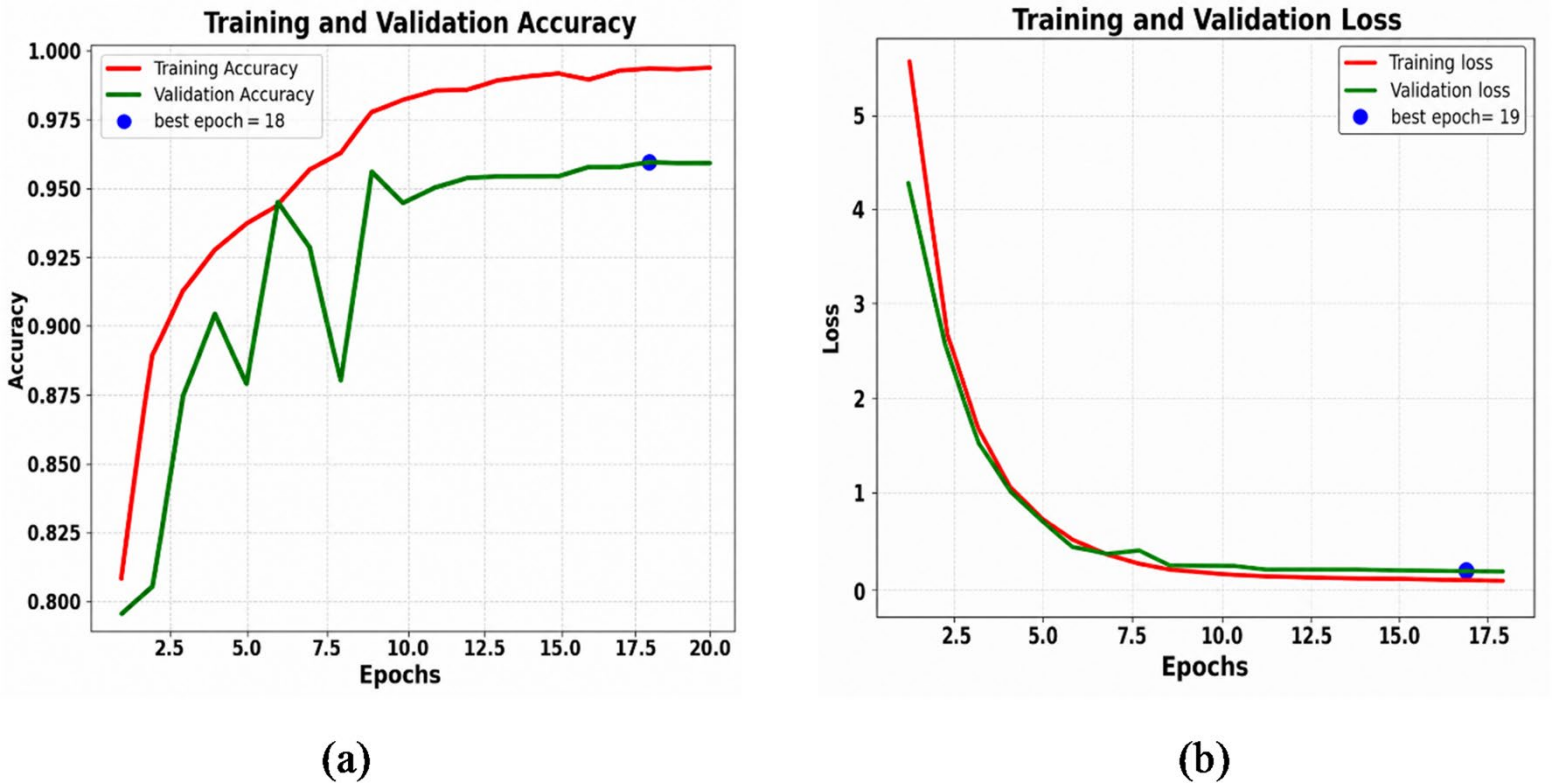
Performance curves of EfficientNetB5: (**a**) accuracy, (**b**) loss.

**Fig. 11:**
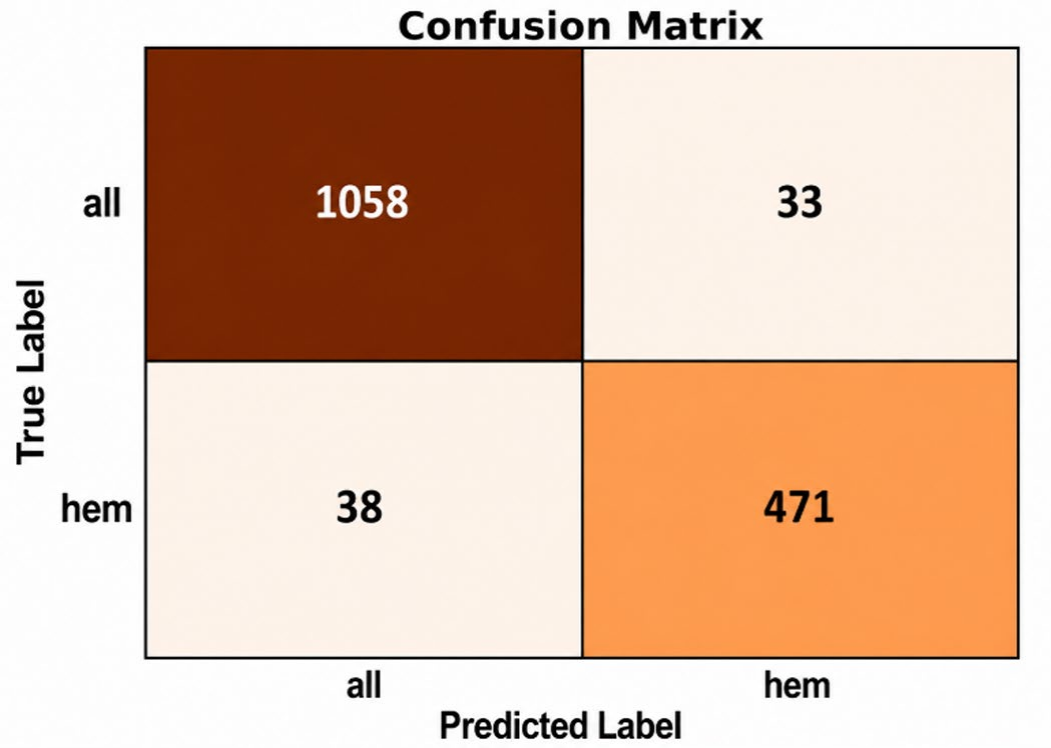
Confusion matrix for EfficientNetB5.

Figure 10 illustrates the training performance of the EfficientNetB5 model, where subfigure (a) presents the accuracy curve and subfigure (b) shows the loss curve during training and validation. EfficientNetB5 achieved a training loss of 0.107, a validation loss of 0.242, and a test loss of 0.242, corresponding to 100% training accuracy, 95.40% validation accuracy, and 95.60% test accuracy. The confusion matrix for the test dataset is shown in Fig. 11, indicating 1058 true positives (TP), 471 true negatives (TN), 38 false positives (FP), and 33 false negatives (FN).

**Fig. 12:**
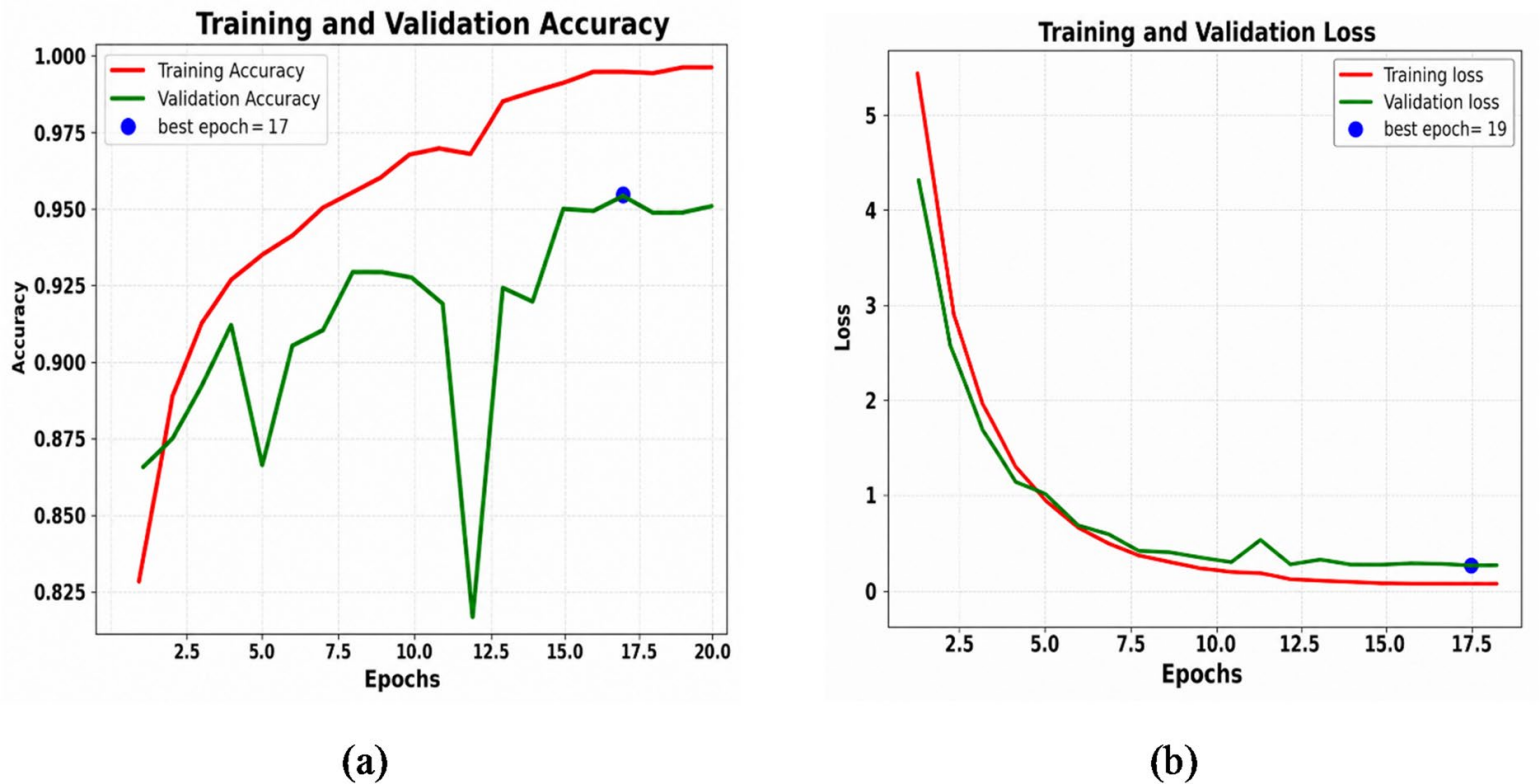
Performance curves of InceptionResNetV2: (**a**) accuracy, (**b**) loss.

**Fig. 13:**
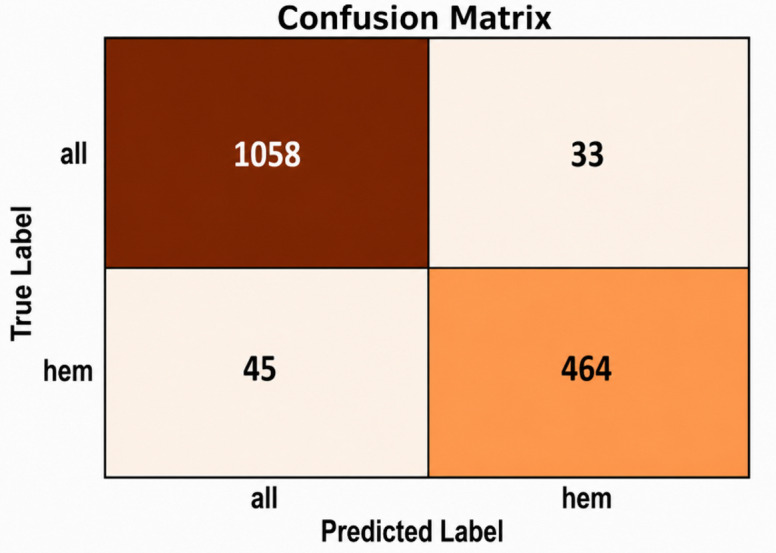
Confusion matrix of InceptionResNetV2.

Figure 12 illustrates the training performance of the InceptionResNetV2 model, where subfigure (a) presents the accuracy curve and subfigure (b) shows the loss curve during the training and validation phases. The model achieved a training loss of 0.112, a validation loss of 0.267, and a test loss of 0.336, corresponding to training, validation, and test accuracies of 100%, 95.50%, and 95.13%, respectively. The confusion matrix for the test dataset is shown in Fig. 13, indicating 1058 true positives (TP), 464 true negatives (TN), 45 false positives (FP), and 33 false negatives (FN). These results demonstrate the capability of the InceptionResNetV2 model to effectively distinguish between leukemic and normal blood cells in microscopic blood smear images.

**Fig. 14:**
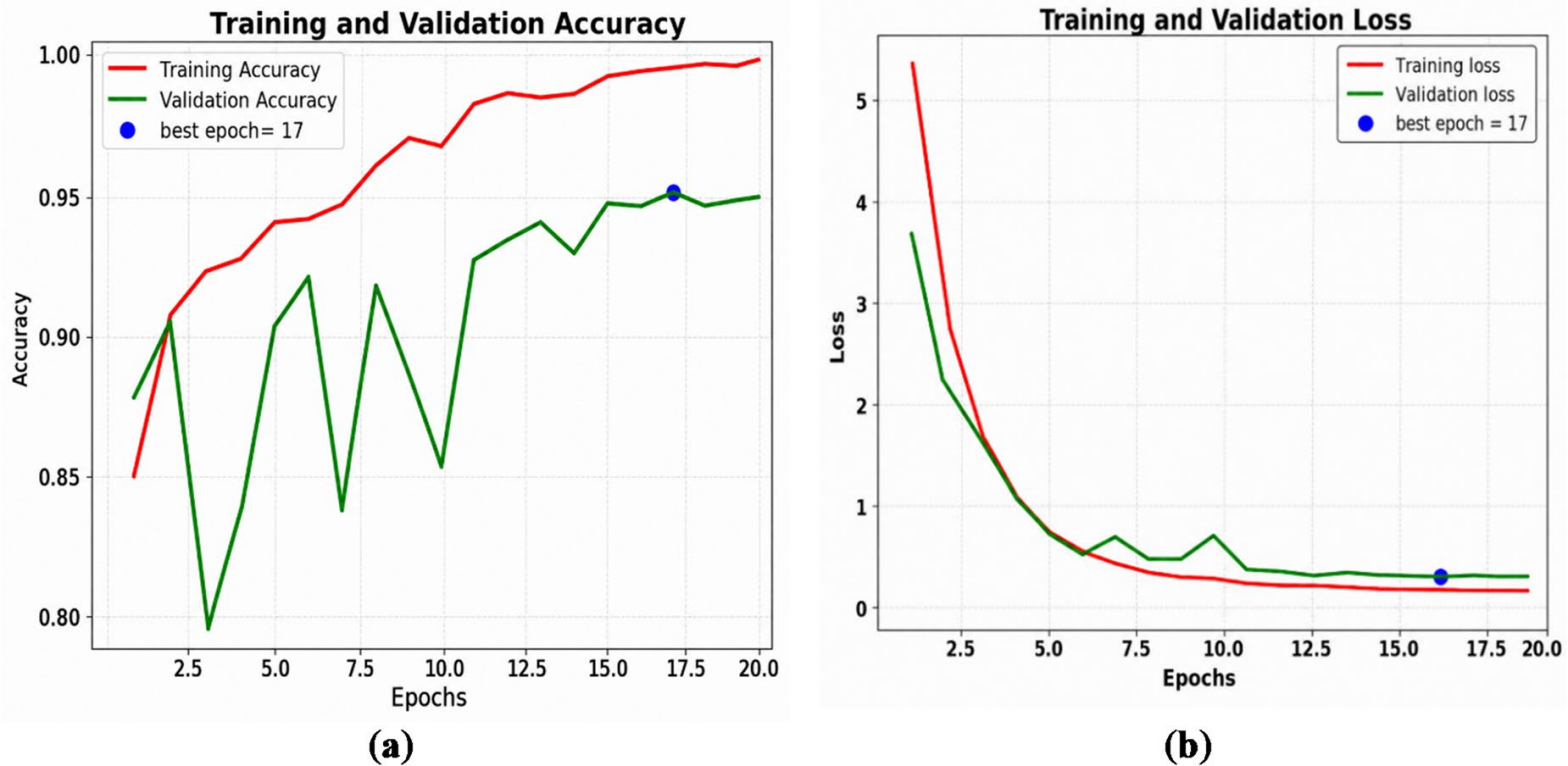
Performance curves of InceptionV3: (**a**) accuracy, (**b**) loss.

**Fig. 15:**
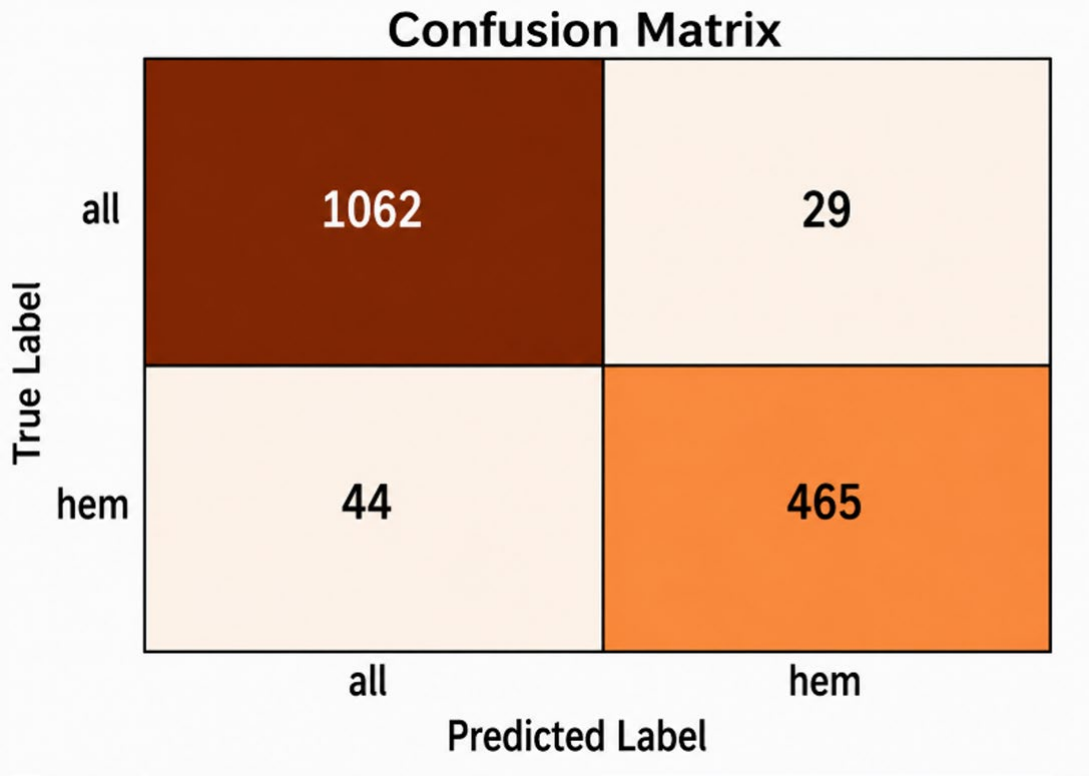
Confusion matrix for InceptionV3.

Figure 14 illustrates the training performance of the InceptionV3 model, where subfigure (a) presents the accuracy curve and subfigure (b) shows the loss curve during the training and validation phases. The model achieved a training loss of 0.097, a validation loss of 0.188, and a test loss of 0.227, corresponding to training, validation, and test accuracies of 99.87%, 96.75%, and 95.45%, respectively. The confusion matrix for the test dataset is shown in Fig. 15, reporting 1062 true positives (TP), 465 true negatives (TN), 44 false positives (FP), and 29 false negatives (FN). These results demonstrate the ability of the InceptionV3 model to effectively distinguish leukemic cells from normal blood cells in microscopic blood smear images.

MobileNet achieved 100% training accuracy, 93.62% validation accuracy, and 93.62% test accuracy, with a train loss of 0.111, validation loss of 0.275, and test loss of 0.292. The accuracy and loss graph for the MobileNet model is shown in Figure S1 (Supplementary materials). The confusion matrix during testing is displayed in Figure S2. The predicted images for True-Positive (TP), True-Negative (TN), False-Positive (FP), and False-Negative (FN) are thus generated in the following order: 1045:453:56:46.

ResNet50 had a training accuracy of 100%, validation accuracy of 94.88%, and test accuracy of 95.94% with a train loss of 0.138, validation loss of 0.292, and test loss of 0.256. The graph of accuracy and loss for the ResNet50 model is shown in Figure S3. The confusion matrix during testing is displayed in Figure S4. The confusion matrix yielded 1066 true positives (TP), 469 true negatives (TN), 40 false positives (FP), and 25 false negatives (FN).

ResNet50V2 demonstrated 92.77% test accuracy, 93% train accuracy, and 92.87% validation accuracy with 0.224 train loss, 0.308 validation loss, and 0.319 test loss. The accuracy and loss graph of the ResNet50V2 model is shown in Figure S5. The confusion matrix during testing is displayed in Figure S6. The predicted images for True- Positive (TP), True-Negative (TN), False-Positive (FP), and False-Negative (FN) are thus generated in the following order: 1043:443:66:48.

With 0.337 train loss, 0.674 validation loss, and 0.440 test loss, ResNet152 demonstrated 96.13% train accuracy, 91.25% validation accuracy, and 92.94% test accuracy. The loss and accuracy graph for the ResNet152 model is shown in Figure S7. The confusion matrix during the test phase is displayed in Figure S8. As a result, the following sequence of predicted images is produced: 1077:410:14:99 for True-Positive (TP), True-Negative (TN), False-Negative (FN), and False-Positive (FP).

With 0.261 train loss, 0.330 validation loss, and 0.346 test loss, ResNet152V2 achieved 96.75% training accuracy, 92.12% validation accuracy, and 92% test accuracy. The ResNet152V2 model’s loss and accuracy graph is shown in Figure S9. The confusion matrix during the test phase is depicted in Figure S10. Thus, for True-Positive (TP), True-Negative (TN), False-Positive (FP), and False-Negative (FN), in that order, 1021:451:58:70 number of predicted images are generated.

With 0.282 train loss, 0.294 validation loss, and 0.283 test loss, VGG16 demonstrated 92.50% of the train, 92.62% of validation accuracy, and 91.81% of test accuracy. The VGG16 model’s loss and accuracy graph is shown in Figure S11. Figure S12 shows the confusion matrix during the testing phase. Thus, for True-Positive (TP), True-Negative (TN), False-Negative (FN), and False-Positive (FP) in that order, 1059:410:32:99 number of predicted images are generated.

VGG19 showed 93.62% of the train, 92% of validation, and 92.06% of test accuracy with 0.270 train loss, 0.302 validation loss, and 0.305 test loss. Figure S13 displays the accuracy and loss for the VGG19 model. Figure S14 shows the confusion matrix during the test phase. Therefore, 1051:422:40:87 number of predicted images are generated for True-Positive (TP), True-Negative (TN), False-Negative (FN), and False-Positive (FP), in that order.

Xception demonstrated a 0.067 train loss, 0.188 validation loss, and 0.195 test loss while displaying 100% of the train, 95.88% of validation, and 95.69% of test accuracy. Figure S15 displays the Xception model’s accuracy and loss chart. The confusion matrix during the test phase is shown in Figure S16. For each of the following, 1067:464:24:45 predicted images are generated in that order: True-Positive (TP), False-Positive (FP), False-Negative (FN), and True-Negative (TN).

Based on the comparative analysis, all evaluated models achieved training, validation, and test losses below 0.250, 0.300, and 0.350, respectively, indicating stable and reliable performance. Notably, DenseNet201, EfficientNetB3, and InceptionV3 exhibited over 99.70% training accuracy and over 96% validation accuracy. Similarly, EfficientNetB1, EfficientNetB5, Xception, InceptionResNetV2, MobileNet, and ResNet50 achieved validation accuracy exceeding 95% with 100% training accuracy. These models also outperformed previously reported studies in terms of precision, recall, and F1-score. Therefore, they are identified as the most effective deep learning architectures for the accurate classification of leukemic and normal cells in microscopic blood smear images.

Based on the proposed models, the results demonstrate improved accuracy and overall performance compared with most previously reported prediction models. Still, there is some room for researchers to improve results. Our proposed work can help researchers and physicians with real-life experience and computer-aided diagnosis. The proposed deep learning models demonstrate promising potential for computer-aided leukemia detection from microscopic images. However, further validation using independent multi-center datasets, along with integration into clinical workflows and compliance with regulatory standards, will be necessary before real-world clinical deployment.

It is important to critically examine the near-perfect accuracy values (approaching or reaching 100%) reported both in several existing studies^45,41–43^ and by some models in the present work. While such results may appear promising, they should be interpreted with considerable caution due to several factors. First, extremely high training accuracy combined with a noticeable gap relative to validation and test accuracy, as observed in this study, may indicate a degree of overfitting, where the model memorizes training samples rather than learning generalizable features. Second, several existing studies reporting 100% accuracy have employed relatively small datasets (ranging from 108 to 3256 images), which substantially increases the likelihood of overfitting and limits the statistical reliability of the reported performance. Models trained on small datasets can easily achieve perfect classification without truly learning discriminative morphological features. Third, the absence of rigorous evaluation protocols, such as k-fold cross-validation, external test sets from independent institutions, or patient-level data splitting, further inflates reported accuracy by allowing potential data leakage or favorable train-test splits. Fourth, binary classification tasks (leukemic vs. normal) are inherently simpler than multi-class subtype classification (e.g., ALL, AML, CLL, CML), and high accuracy in binary settings does not necessarily translate to robust multi-class performance. In the present study, although the best-performing models achieved up to 100% training accuracy, the test accuracy consistently ranged between 95% and 96%, reflecting a realistic and more conservative performance estimate. Furthermore, all experiments were repeated ten times using different random seeds, and performance metrics are reported as mean values across these runs to ensure statistical robustness. Additionally, pairwise McNemar’s tests were conducted to confirm that the observed performance differences between models were statistically significant. Future work should further strengthen validation by incorporating multi-center datasets, patient-wise data splitting, k-fold cross-validation, and external clinical testing to provide a more rigorous assessment of model generalizability and clinical readiness.

### Overfitting and regularization

During training, several models (notably EfficientNetB1, EfficientNetB5, Xception, InceptionResNetV2, MobileNet, and ResNet50) achieved near-perfect training accuracy approaching 100%, while their validation and test accuracies remained consistently in the range of 93 to 96%. We respectfully clarify that this behavior, while warranting careful interpretation, does not indicate severe overfitting, and is supported by four pieces of empirical evidence. First, the train-test accuracy gap is bounded between 4 and 7%, which is within the acceptable range for transfer learning on moderately sized medical imaging datasets^38,39,45^. Second, examination of the validation-loss curves (Figs. 4, 6, 8, 10, 12 in the main manuscript and Figures S1, S3, and S15 in the Supplementary Information) shows that the validation loss did not exhibit a consistent upward trend during later epochs, indicating that the models were not progressively memorizing training data, which is the hallmark behavior of severe overfitting. Third, this pattern is commonly observed when powerful ImageNet pre-trained networks are fine-tuned on relatively well-separated binary classification tasks, because the pre-trained convolutional features are already highly discriminative and require minimal adaptation. Fourth, the stability of performance across ten repeated runs with different random seeds, evidenced by the low standard deviation across all metrics, further confirms that the high training accuracy is not an artifact of a single favorable data split, but a consistent and reproducible characteristic of the trained models. Together, these observations support the conclusion that the observed near-perfect training accuracy reflects the strong feature transferability of pre-trained ImageNet weights combined with effective regularization, rather than uncontrolled memorization. Nevertheless, we agree that 100% training accuracy remains a cautionary indicator, and accordingly the regularization strategies described in the next paragraph were specifically employed to bound generalization error throughout training.

To mitigate potential overfitting, multiple regularization strategies were employed: (1) data augmentation techniques, including rotation, flipping, shifting, and zooming, were applied to increase dataset variability; (2) dropout with a rate of 0.5 was incorporated in the fully connected classification layers to reduce neuron co-adaptation; and (3) early stopping was implemented by monitoring the validation loss with a patience value of 5 epochs, automatically terminating training when no further improvement was observed. Collectively, these techniques contributed to maintaining stable and consistent performance across repeated experimental runs.

To quantitatively analyze the extent of overfitting, two standard indicators were computed for all fourteen evaluated architectures: the generalization gap (defined as train accuracy minus test accuracy) and the train-validation loss gap, both extracted from the values reported in Table 4 and the corresponding training curves. The generalization gap ranged from approximately 0.7% (VGG16) to 6.4% (MobileNet), with a mean of approximately 4.3% across all models. For the four best-performing models (EfficientNetB1, EfficientNetB5, Xception, ResNet50), the generalization gap was bounded between 3.94% and 4.40%, and the corresponding train-validation loss gap remained between 0.10 and 0.16. According to widely adopted thresholds in transfer-learning literature, a generalization gap below approximately 5% is considered indicative of acceptable generalization, gaps between 5 and 10% indicate mild overfitting that warrants regularization, and gaps exceeding 10% indicate severe overfitting. Under this criterion, all fourteen evaluated architectures fall within or marginally above the acceptable-generalization range, with no model exhibiting severe overfitting. Furthermore, the standard deviation of the generalization gap across the ten repeated runs remained below 1.0% for all top-performing models, indicating that this bounded gap is a stable property of the trained models rather than a random-split artifact. These quantitative results empirically confirm that the regularization strategies (data augmentation, dropout, and early stopping) effectively bounded overfitting throughout the experimental pipeline, and that the reported test-set performance is a reliable indicator of generalization capability rather than a product of training-set memorization.

An additional concern related to model reliability is the potential for data leakage due to the absence of patient-level identifiers in the dataset. Without such metadata, images from the same patient may appear in both the training and test sets, potentially allowing the model to learn patient-specific features rather than generalizable morphological patterns, thereby inflating reported performance. To partially mitigate this risk, stratified random sampling, data augmentation, and repeated experiments with different random seeds and data splits were employed. The consistent performance with low standard deviations across repeated runs provides indirect evidence that the models are not excessively dependent on any particular data partition. Nevertheless, these strategies cannot fully substitute for true patient-wise splitting. Future work will address this through evaluation on clinically curated datasets with patient-level annotations, implementation of Leave-One-Patient-Out (LOPO) cross-validation, and external validation on independent multi-center datasets to rigorously quantify and eliminate the impact of potential data leakage.

Specifically, because patient identifiers are unavailable in the dataset, patient-wise data splitting could not be implemented, and the possibility of patient-level overlap between the training, validation, and test subsets cannot be fully excluded. This represents a recognized source of potential data leakage that may slightly inflate the reported performance metrics. While the mitigation strategies described above (stratified sampling, data augmentation, repeated runs with different random seeds, low standard deviation across runs, and McNemar’s significance testing) reduce, but do not eliminate, this risk, full elimination of patient-level data leakage will require subject-wise partitioning protocols on clinically annotated datasets. Accordingly, patient-stratified validation, including Leave-One-Patient-Out (LOPO) cross-validation and prospective evaluation on independent multi-center clinical datasets, is committed to as the highest-priority direction for subsequent work, and the present results should be interpreted as architectural benchmarking outcomes rather than as definitive clinical performance estimates.

Despite the application of these regularization methods, the gap between training and test accuracy suggests that minor overfitting may still persist, particularly for deeper architectures. In future work, additional mitigation techniques such as L2 weight regularization, batch normalization, k-fold cross-validation, and model ensembling can be integrated to further improve generalization and reduce variance. Incorporating explainable AI (XAI) mechanisms alongside these techniques will also help ensure reliable predictions while maintaining transparency for clinical decision-making.

### Comparison with proposed work

To contextualize the proposed work within the existing literature, a comparative analysis with previously published studies is presented in Table 8. We respectfully emphasize, however, that this comparison is not statistically rigorous and must be interpreted with substantial caution, because direct comparison across studies is fundamentally limited by several methodological differences. Specifically: (i) dataset differences—previous studies use datasets of widely varying sizes (108 to 15,135 images), with different class distributions, image acquisition devices, staining protocols, and patient populations, all of which materially affect reported accuracy; (ii) task definition differences—some studies address binary classification (leukemic vs. normal), while others address multi-class classification (e.g., ALL subtypes, AML detection, or four-class FAB-style categorization), which are inherently different problems and not directly comparable on a single accuracy figure; (iii) preprocessing and augmentation differences—different studies apply different resizing strategies, normalization schemes, and augmentation pipelines, all of which influence training dynamics and reported results; (iv) training-protocol differences—variations in optimizers, learning rates, batch sizes, training epochs, regularization techniques, and hardware platforms further confound direct comparison; (v) statistical reporting differences—most previous studies report results from a single experimental run, whereas this study reports mean ± standard deviation across ten repeated runs, making the underlying performance distributions non-equivalent; and (vi) unavailability of raw predictions—confusion matrices and paired predictions from previous studies are not publicly available, which precludes paired statistical testing (e.g., paired t-tests or McNemar’s tests) against external baselines.

For these reasons, Table 8 is intended solely as a contextual benchmarking reference, providing a high-level overview of how the proposed framework relates to recent leukemia detection studies in terms of dataset scale, model diversity, and reported performance, rather than a statistically validated ranking of model superiority. To partially compensate for this limitation, rigorous internal statistical validation was performed within this study: all experiments were repeated ten times with different random seeds, performance metrics are reported as mean ± standard deviation, and pairwise McNemar’s tests confirmed that the performance differences between the fourteen evaluated models are statistically significant at the 95% confidence level (*p* < 0.05). To enable fair external statistical comparison, future work will involve re-implementing selected baseline methods (e.g., from references^36,38,39,44,45^) on the same dataset under identical preprocessing, training, and evaluation protocols, thereby providing paired predictions that support direct McNemar’s or paired t-test comparisons against the proposed framework.

**Table 8:** Comparison of the study with the previously published work.

References of study	Year	Datasets	Classes	Proposed method	Accuracy (%)
Raheel Baig et al.^4^	2022	118 images	3	Bagging ensemble	95.04
K.Dhana Shree et al.^56^	2024	118 images	4	CNN	95.98
Mei et al.^57^	2025	1794 images	3	Spatially-Guided Learning Network (SGLNet) based on YOLOv8 with SGB, SAF, CBAM, and EIoU	95.9
Saikia et al.^58^	2025	1600 + 1718 images	2	VNLU-Net (VGG16 with Lightweight Union-Net module)	99.71
Soladoye et al.^39^	2025	10,661 images	2	Deep Transfer Learning using EfficientNet-B3 and VGG-19	96
Das et al.^59^	2025	108 + 260 images	2	Hybrid transfer learning framework combining MobileNetV2 and ShuffleNet	99.07
Pervez et al.^60^	2025	15,135 images	2	Hierarchical Federated Learning with EfficientNetB3 and Explainable AI (Saliency, Occlusion, RISE)	96.5
Basaran et al.^61^	2025	3256 images	4	Hybrid model: Grad-CAM + DarkNet19 + AutoEncoder + Harris Hawk Optimization (HHO) with kNN classifier	98.52
Proposed work	2025	10,700 images	2	EfficientNetB1, EfficientNetB5, Xception, ResNet50	Train: 100 Validation: 96.50 Test: 96

Overall, the comparative analysis reveals that advanced deep transfer learning models, particularly EfficientNetB1, EfficientNetB5, Xception, and ResNet50, achieved superior performance in classifying leukemia from microscopic images. These models demonstrated high accuracy, low loss, and a strong precision-recall balance, indicating their potential as candidate tools for future computer-aided diagnostic support. While the results provide promising evidence regarding the effectiveness of automated deep learning approaches, full clinical integration in hematological diagnosis will require external validation on independent multi-center clinical datasets, prospective evaluation in real diagnostic workflows, and compliance with applicable regulatory standards.

To further substantiate this claim, a detailed quantitative comparison with recent studies is warranted. For instance, Nasir et al. (2023)^36^ achieved 97.3% accuracy using AlexNet but evaluated only three models on a comparatively smaller dataset. Haque et al. (2024)^37^ reported an F1-score of 96.07% using Inception-ResNet for binary classification, yet their study was limited to a restricted number of architectures. Kumar et al. (2025)^38^ achieved 98.59% validation accuracy with InceptionResNetV2; however, their framework was constrained by non-uniform image sizes and limited model diversity. Muduli et al. (2025)^45^ reported up to 100% accuracy using VGG16 and ResNet50, but acknowledged the risk of overfitting due to a small dataset. In contrast, the present study provides a more comprehensive benchmarking framework by evaluating fourteen CNN architectures on a substantially larger dataset of 10,700 images, achieving consistently high accuracy (up to 96%), recall (up to 98%), and F1-scores (up to 97%) across the best-performing models. Furthermore, unlike the majority of the aforementioned studies, this work uniquely integrates Explainable AI (XAI) techniques, including Grad-CAM and LIME, to enhance model interpretability and clinical trustworthiness. This combination of extensive model comparison, large-scale dataset evaluation, and explainability analysis collectively provides stronger justification for the superiority of the proposed framework.

The growing body of recent literature (2023–2025) further supports the relevance of this study. While recent works such as Pervez et al. (2025)^60^ and Muduli et al. (2025)^45^ have begun integrating explainable AI into leukemia detection frameworks, these studies typically evaluate a narrow set of CNN architectures. The present study addresses this gap by providing one of the most extensive comparative evaluations reported to date, systematically benchmarking fourteen architectures under identical experimental conditions with integrated XAI analysis^62^.

Although classification performance in this study was evaluated using accuracy, precision, recall, and F1-score, calibration analysis of predicted probabilities was not included. Calibration techniques, such as reliability diagrams or Brier scores, can provide additional insight into how well the predicted probabilities reflect true outcome likelihoods, which is particularly important for clinical decision-support systems. Incorporating such evaluation methods can improve the interpretability and trustworthiness of model predictions in medical applications. Future research will therefore integrate calibration assessment techniques to further examine the reliability of probabilistic outputs.

### Clinical interpretation of the findings

Beyond the technical performance metrics, the results of this study carry several clinically meaningful implications that warrant deeper interpretation. First, the high recall (up to 98%) and sensitivity (approximately 98% at 95% specificity, as observed in the ROC analysis) achieved by the top-performing models, particularly EfficientNetB1 and ResNet50, indicate that the proposed framework would, in principle, miss very few leukemic cases if deployed as a screening tool — a property that is especially important in hematological diagnostics, where missed leukemia cases can have severe and irreversible clinical consequences. Second, the false-positive rate of approximately 2 to 5% observed across the top-performing architectures, while acceptable for an initial screening stage, would translate into additional confirmatory testing (bone-marrow aspiration, flow cytometry, immunophenotyping, or cytogenetic analysis) for a small subset of patients; this is consistent with how computer-aided diagnostic systems are typically positioned in real clinical workflows, namely as triage and decision-support tools rather than autonomous diagnostic systems. Third, the Grad-CAM and LIME outputs carry direct clinical value because they enable hematopathologists to verify that the models attend to morphologically meaningful regions (abnormal nuclei, irregular nucleus-to-cytoplasm ratios, chromatin clumping, and cytoplasmic granularity) that are the same features routinely used in manual French American British (FAB) and World Health Organization (WHO) classification of leukemia, thereby aligning the model’s reasoning with established hematological diagnostic criteria. Fourth, the inference latency of 20 to 35 milliseconds per image is well below the clinically practical threshold for real-time decision support, suggesting that integration into digital pathology workflows and laboratory information systems is technically feasible without disrupting routine slide-review timelines. Fifth, despite these promising indicators, the study’s binary classification scope (leukemic vs. normal) means that the present framework cannot, on its own, distinguish between clinically distinct leukemia subtypes such as ALL, AML, CLL, and CML, each of which has different prognoses and treatment pathways; multi-class classification is therefore an essential next step before clinical translation. Sixth, the absence of patient-level metadata, external validation, calibration of predicted probabilities, and prospective clinical trial evidence collectively imply that the present results should be interpreted as technically promising but not yet clinically actionable; deployment in real diagnostic settings will require external multi-center validation, regulatory approval (e.g., FDA, EMA, or BMDC pathways), integration testing within hospital information systems, prospective clinical trials, and explicit clinician-in-the-loop oversight protocols.

In clinical practice, the interpretation of microscopic blood smear images may vary among hematologists and pathologists due to differences in experience and subjective assessment of cellular morphology. This inter-observer variability represents a well-known challenge in hematological diagnostics and highlights the need for computer-aided diagnostic systems capable of providing consistent decision support. However, the publicly available dataset used in this study does not include detailed information regarding the annotation process or the level of agreement among expert annotators. Future studies will aim to evaluate the proposed models using clinically curated datasets with expert consensus annotations that follow established hematological diagnostic standards. Although Grad-CAM was incorporated as an interpretability mechanism, detailed visualization and quantitative analysis of the attention regions were beyond the scope of this comparative benchmarking study. Future work will include representative Grad-CAM heatmaps and cell-level interpretability analysis to further improve model transparency and clinical interpretability.

A major limitation of this study is the absence of external validation and cross-dataset testing on independent benchmark datasets. All models were trained and evaluated on a single publicly available dataset, and although ten repeated experiments with different random seeds, stratified sampling, and pairwise McNemar’s significance testing were employed to enhance internal robustness, these strategies do not substitute for validation on unseen data acquired from different sources. External validation is essential to assess whether the learned features generalize across variations in imaging devices, staining protocols (Wright, Giemsa, May Grünwald Giemsa), magnification levels, illumination conditions, and patient populations from different clinical institutions. Without such validation, the reported performance metrics may not fully reflect real-world clinical applicability. It is worth noting that this limitation is shared by the majority of existing studies in the leukemia detection literature^36–39,44,45^, where models are typically evaluated on a single dataset without cross-dataset testing. To address this in subsequent work, a structured cross-dataset evaluation protocol is committed to, comprising: (i) evaluation on multiple independent benchmark datasets, including ALL-IDB1, ALL-IDB2, C-NMC 2019, ASH image datasets, and Munich AML datasets; (ii) leave-one-dataset-out validation, in which the models are trained on a subset of datasets and evaluated on the held-out dataset to quantify cross-source generalization; (iii) domain adaptation strategies, including stain normalization (Macenko, Reinhard, Vahadane), adversarial domain adaptation, and style-transfer augmentation, to bridge cross-dataset distribution shifts; and (iv) prospective evaluation on clinically curated multi-center data from collaborating medical institutions with patient-level annotations and expert-consensus labels. This cross-dataset evaluation protocol will provide rigorous evidence of generalizability, robustness, and clinical readiness of the proposed benchmarking framework, beyond the internal-validation results reported in this study.

It is therefore emphasized that the term “clinical applicability”, as used in this study, refers to the potential of the proposed framework to inform future clinical decision-support systems, rather than to immediate readiness for deployment in real clinical environments. The reported performance metrics are obtained from a single publicly available dataset, and external validation on prospectively collected, clinically annotated, and multi-institutional datasets is essential before any operational clinical use. Accordingly, all clinical-related claims throughout this manuscript are intended in the sense of “preliminary evidence supporting future clinical translation”, contingent on subsequent external validation, regulatory approval, and integration testing within established hematological diagnostic workflows.

## Conclusion

This study presented a comprehensive comparative benchmarking of fourteen pre-trained deep transfer learning architectures for binary leukemia detection from microscopic blood smear images, using a publicly available dataset of 10,700 labeled samples. The best-performing architectures, including EfficientNetB1, EfficientNetB5, Xception, and ResNet50, achieved test accuracies in the range of 95 to 96%, with corresponding AUC values up to 0.987 and consistently strong precision, recall, and F1-scores. Methodologically, the study contributes a unified, reproducible benchmarking framework that integrates dual Explainable AI techniques (Grad-CAM and LIME), repeated-run statistical validation with pairwise McNemar’s testing, deployment-aware computational profiling, and quantitative interpretability evaluation. These elements collectively distinguish the present work as a methodologically rigorous reference benchmark rather than a proposal of a novel architecture.

However, the reported results should be interpreted with appropriate caution and must not be construed as evidence of clinical readiness. Several material limitations constrain the strength of the conclusions that can be drawn. First, the study relies on a single publicly available binary-class dataset that lacks patient-level metadata, preventing subject-wise data partitioning and introducing a recognized risk of data leakage that internal mitigation strategies (stratified sampling, augmentation, repeated runs, and McNemar’s testing) can reduce but not eliminate. Second, the absence of external validation and cross-dataset testing means that the reported performance metrics may not reflect real-world generalization across imaging devices, staining protocols, magnification levels, and patient populations from different clinical institutions. Third, the framework is restricted to binary classification and cannot, on its own, distinguish between clinically distinct leukemia subtypes (ALL, AML, CLL, CML), each of which has different prognostic and therapeutic implications. Fourth, the study uses uniform hyperparameters and a fixed training schedule across all architectures, which, although a deliberate methodological safeguard for fair comparison, may have led to undertraining of the deepest models such as ResNet152 and ResNet152V2. Fifth, calibration of predicted probabilities, formal augmentation ablation studies, and prospective clinician-in-the-loop evaluation were beyond the scope of the present benchmarking study.

Accordingly, the present results should be interpreted as preliminary benchmarking evidence supporting the future development of computer-aided leukemia detection tools, rather than as evidence supporting immediate clinical deployment. To bridge this gap, future work will pursue a structured research program comprising: (i) multi-class classification of leukemia subtypes (ALL, AML, CLL, CML) using clinically annotated datasets; (ii) cross-dataset validation on independent benchmarks (ALL-IDB1, ALL-IDB2, C-NMC 2019, ASH image datasets, and Munich AML datasets) and prospective multi-center clinical data with patient-level annotations; (iii) patient-wise data splitting and Leave-One-Patient-Out (LOPO) cross-validation to eliminate residual data leakage; (iv) architecture-specific hyperparameter optimization (Bayesian, grid, and population-based) and systematic ablation studies for augmentation and training strategies; (v) calibration analysis (reliability diagrams, Brier scores, temperature scaling) to ensure trustworthy probabilistic outputs; (vi) domain adaptation strategies, including stain normalization (Macenko, Reinhard, Vahadane), adversarial domain adaptation, and style-transfer augmentation, to improve robustness across institutions; (vii) comparative experimental evaluation against Vision Transformer and hybrid CNN-Transformer architectures under identical experimental conditions; and (viii) prospective clinical trials with clinician-in-the-loop deployment, regulatory compliance, and integration testing within hospital information systems prior to any operational clinical use. Within these scoped boundaries, the unified benchmarking framework presented in this study is intended to serve as a reproducible reference foundation that supports both methodological research and the eventual translation of deep learning-based leukemia detection into validated clinical practice.

## Supplementary Information

Below is the link to the electronic supplementary material.

Supplementary Material 1

## Data Availability

The dataset used in this study is publicly available at: https://www.kaggle.com/datasets/andrewmvd/leukemia-classification.

## Code availability

The complete implementation, including the training scripts, model configuration files, augmentation pipeline, evaluation routines, Grad-CAM and LIME visualization scripts, and hyperparameter settings used in this study, will be made publicly available in a dedicated GitHub repository upon acceptance of the manuscript. Until that point, all implementation details, training configurations, and reproducibility-related materials are available from the corresponding author upon reasonable request. The publicly available dataset used in this study is accessible at https://www.kaggle.com/datasets/andrewmvd/leukemia-classification.
