# Supplementary material for: Automated leukemia detection from microscopic images using deep transfer learning with explainable AI-based analysis
Source: Sci Rep. 2026 May 30;16:24693. doi: 10.1038/s41598-026-55415-x (PMC13454600; doi:10.1038/s41598-026-55415-x)
Supplement: Supplementary file 1 [file 41598_2026_55415_MOESM1_ESM.docx]

**Supplementary Information**

for

**Manuscript Title:** Automated Leukemia Detection from Microscopic Images Using Deep Transfer Learning: A Comprehensive Comparative and Explainable AI–Based Analysis of CNN Architectures

**Authors:** Md Ashikuzzaman, Mir Md. Julhash, Md. Feroz Ali, Md Shafiul Alam, Mohammad Ali, Imil Hamda Imran, Obaidullah Obaidi, and Md Kamrul Islam

Table of Contents

| **Figure No.** | **Title** |
| --- | --- |
| Figure S1 | Accuracy and Loss Graph of MobileNet |
| Figure S2 | Confusion Matrix of MobileNet |
| Figure S3 | Accuracy and Loss Graph of ResNet50 |
| Figure S4 | Confusion Matrix of ResNet50 |
| Figure S5 | Accuracy and Loss Graph of ResNet50V2 |
| Figure S6 | Confusion Matrix of ResNet50V2 |
| Figure S7 | Accuracy and Loss Graph of ResNet152 |
| Figure S8 | Confusion Matrix of ResNet152 |
| Figure S9 | Accuracy and Loss Graph of ResNet152V2 |
| Figure S10 | Confusion Matrix of ResNet152V2 |
| Figure S11 | Accuracy and Loss Graph of VGG16 |
| Figure S12 | Confusion Matrix of VGG16 |
| Figure S13 | Accuracy and Loss Graph of VGG19 |
| Figure S14 | Confusion Matrix of VGG19 |
| Figure S15 | Accuracy and Loss Graph of Xception |
| Figure S16 | Confusion Matrix of Xception |

In terms of accuracy, this MobileNet demonstrated 100% of the train, 93.62% of validation, and 93.62% of the test, with a train loss of 0.111, validation loss of 0.275, and test loss of 0.292. The accuracy and loss graph for the MobileNet model is shown in Figure S1. The confusion matrix during testing is displayed in Figure S2. The predicted images for True-Positive (TP), True-Negative (TN), False-Positive (FP), and False-Negative (FN) are thus generated in the following order: 1045:453:56:46.

(a)	(b)

Figure S1. Accuracy and Loss graph of Mobile-Net.

Figure S2. Confusion Matrix for MobileNet.

ResNet50 had a training accuracy of 100%, validation accuracy of 94.88%, and test accuracy of 95.94% with a train loss of 0.138, validation loss of 0.292, and test loss of 0.256. The graph of accuracy and loss for the ResNet50 model is shown in Figure S3. The confusion matrix during testing is displayed in Figure S4. To generate the predicted images for True-Positive (TP), True-Negative (TN), False-Positive (FP), and False-Negative (FN), 1066:469:40:25 are generated.

(a)	(b)

Figure S3. Accuracy and Loss graph of ResNet50.

Figure S4. Confusion Matrix of ResNet50.

ResNet50V2 demonstrated 92.77% test accuracy, 93% train accuracy, and 92.87% validation accuracy with 0.224 train loss, 0.308 validation loss, and 0.319 test loss. The accuracy and loss graph of the ResNet50V2 model is shown in Figure S5. The confusion matrix during testing is displayed in Figure S6. The predicted images for True- Positive (TP), True-Negative (TN), False-Positive (FP), and False-Negative (FN) are thus generated in the following order: 1043:443:66:48.

(a)	(b)

Figure S5. Accuracy and Loss graph of ResNet50V2.

Figure S6. Confusion Matrix of ResNet50V2.

With 0.337 train loss, 0.674 validation loss, and 0.440 test loss, ResNet152 demonstrated 96.13% train accuracy, 91.25% validation accuracy, and 92.94% test accuracy. The loss and accuracy graph for the ResNet152 model is shown in Figure S7. The confusion matrix during the test phase is displayed in Figure S8. As a result, the following sequence of predicted images is produced: 1077:410:14:99 for True-Positive (TP), True-Negative (TN), False-Negative (FN), and False-Positive (FP).

(a)	(b)

Figure S7. Accuracy and Loss graph of ResNet152.

Figure S8. Confusion Matrix of ResNet152.

With 0.261 train loss, 0.330 validation loss, and 0.346 test loss, ResNet152V2 demonstrated 96.75% of the train, 92.12% of validation accuracy, and 92% of test accuracy. The ResNet152V2 model's loss and accuracy graph is shown in Figure S9. The confusion matrix during the test phase is depicted in Figure S10. Thus, for True-Positive (TP), True-Negative (TN), False-Positive (FP), and False-Negative (FN), in that order, 1021:451:58:70 number of predicted images are generated.

(a)	(b)

Figure S9. Accuracy and Loss graph of ResNet152V2.

Figure S10. Confusion Matrix of ResNet152V2.

With 0.282 train loss, 0.294 validation loss, and 0.283 test loss, VGG16 demonstrated 92.50% of the train, 92.62% of validation accuracy, and 91.81% of test accuracy. The VGG16 model's loss and accuracy graph is shown in Figure S11. Figure S12 shows the confusion matrix during the testing phase.Thus, for True-Positive (TP), True-Negative (TN), False-Negative (FN), and False-Positive (FP) in that order, 1059:410:32:99 number of predicted images are generated.

(a)	(b)

Figure S11. Accuracy and Loss graph of VGG16.

Figure S12. Confusion Matrix of VGG16.

VGG19 showed 93.62% of the train, 92% of validation, and 92.06% of test accuracy with 0.270 train loss, 0.302 validation loss, and 0.305 test loss. Figure S13 displays the accuracy and loss for the VGG19 model. Figure S14 shows the confusion matrix during the test phase. Therefore, 1051:422:40:87 number of predicted images are generated for True-Positive (TP), True-Negative (TN), False-Negative (FN), and False-Positive (FP), in that order.

(a)	(b)

Figure S13. Accuracy and Loss graph of VGG19.

Figure S14. Confusion Matrix of VGG19.

Xception demonstrated a 0.067 train loss, 0.188 validation loss, and 0.195 test loss while displaying 100% of the train, 95.88% of validation, and 95.69% of test accuracy. Figure S15 displays the Xception model's accuracy and loss chart. Confusion matrix during the test phase (Figure S16). For each of the following, 1067:464:24:45 predicted images are generated in that order: True-Positive (TP), False-Positive (FP), False-Negative (FN), and True-Negative (TN).

(a)	(b)

Figure S15. Accuracy and Loss graph of Xception.

Figure S16. Confusion Matrix of Xception.
